# Recent progress of gas sensors toward olfactory display development

**DOI:** 10.1186/s40580-025-00508-y

**Published:** 2025-08-29

**Authors:** Ye-Ji Kim, Chae Young Woo, Yeonggwon Kim, Sung Min Kim, Na-Yeong Kim, Hyung Woo Lee, Jin-Woo Oh

**Affiliations:** 1 Humanoid Olfactory Display Innovation Research Center, Pusan National University, Busan, 46241 Republic of Korea; 2 Research Center of Energy Convergence Technology, Pusan National University, Busan, 46241 Republic of Korea; 3 Department of Nano Fusion Technology, Pusan National University, Busan, 46241 Republic of Korea; 4 Institute of Nanobio Convergence, Pusan National University, Busan, 46241 Republic of Korea

**Keywords:** Gas sensors, Olfactory display, Organic material, Inorganic material, Hybrid material, M13 bacteriophage, Carbon nanotube

## Abstract

Olfactory display systems, designed to replicate the human sense of smell, rely on gas sensors that are fast, selective, and reliable. From this perspective, this review highlights recent progress in sensing materials and integration strategies that enable room-temperature operation, rapid response and recovery, and closed-loop control for realistic odor delivery. Advances are classified into three categories: organic, inorganic, and hybrid systems. Organic materials, including conductive polymers and biomolecules, offer tunable selectivity and lightweight flexibility. Inorganic semiconductors, especially metal oxides, provide high sensitivity and durability, though they typically require elevated temperatures. Hybrid architectures, exemplified by M13 bacteriophage–carbon nanotube composites, merge these strengths to achieve superior performance under ambient conditions. Particular emphasis is placed on sensors for ethylene, hydrogen sulfide, hydrogen, acetone, and nitrogen dioxide—gases critical to food preservation, environmental monitoring, and healthcare. Finally, we discuss persistent challenges, such as selectivity under complex conditions, device miniaturization, and closed-loop integration, and propose strategic research directions toward immersive, real-time olfactory display technologies.

**Supplementary Information:**

The online version contains supplementary material available at 10.1186/s40580-025-00508-y.

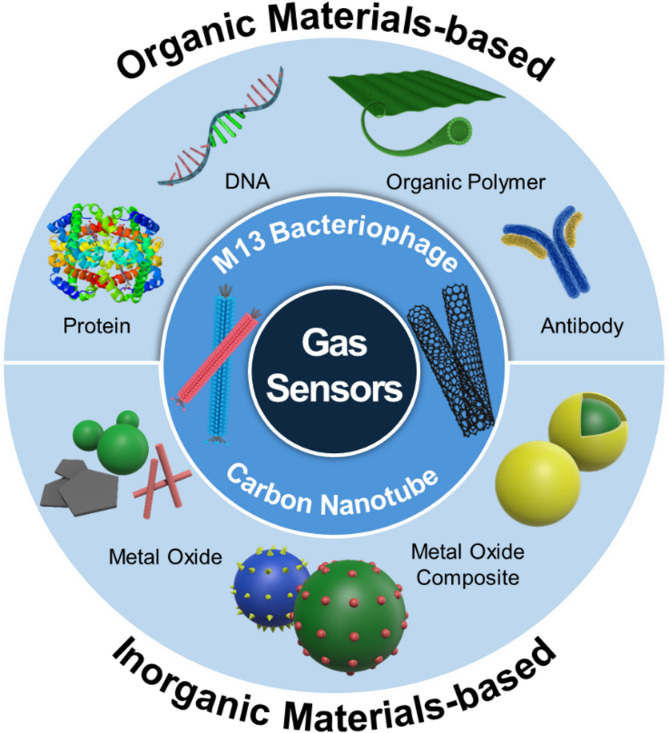

## Introduction

**Fig. 1:**
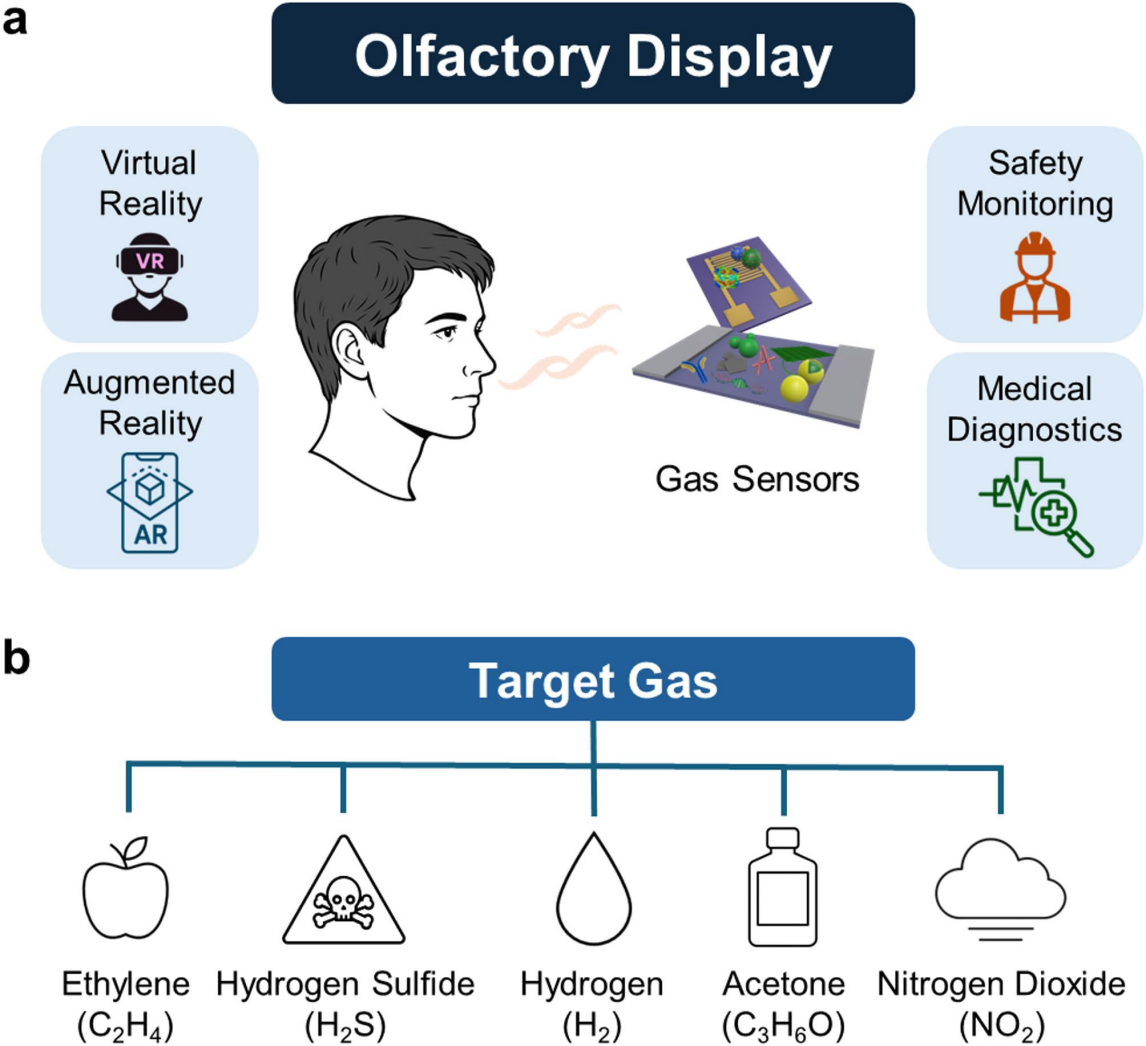
**a** Conceptual Schematic of a gas-sensor-based olfactory display for various applications. **b** Major target gas species in this review paper. This schematic illustrates a real-time olfactory display system, highlighting gas sensors as feedback elements that monitor scent module output and enable precise, closed-loop control of odor delivery

The human sense of smell is a fundamental sensory modality, closely associated with environmental perception, memory, and emotion [1, 2]. Reproducing this complex mechanism in artificial systems has become a major challenge in humanoid robotics. In recent years, the integration of gas sensor technologies into olfactory display systems has drawn significant attention, opening possibilities for applications in virtual reality (VR), augmented reality (AR), safety monitoring, medical diagnostics, and human–robot interaction (HRI) (Fig. 1a) [3–5].

The concept of the electronic nose (e-nose), first introduced in the 1980s [6], provides important historical context. Early e-nose systems relied on metal oxide sensor arrays and simple pattern recognition [7, 8], while modern platforms can classify complex odor profiles using advanced sensor arrays and data processing [9, 10]. However, unlike e-nose devices that primarily focus on odor detection and classification, olfactory display systems require gas sensors to function as real-time feedback elements, enabling precise odor synthesis, modulation, and delivery in immersive environments. Thus, the design requirements for olfactory display sensors—fast response/recovery, closed-loop control, high selectivity, and compatibility with miniaturized, wearable formats—are more stringent than those of conventional e-nose platforms [11–14].

To meet these demands, a wide variety of next-generation sensing materials have been explored. Notably, metal oxide semiconductors (MOS), carbon nanotubes (CNT), graphene, conductive polymers, and hybrid nanostructures such as metal-organic frameworks (MOF) and organic–inorganic composites have demonstrated excellent performance at room temperature, with improvements in both sensitivity and selectivity [15–17]. In parallel, artificial olfaction systems incorporating pattern recognition and machine learning algorithms have enabled real-time classification and contextual odor analysis under complex conditions [18, 19].

Simultaneously, technologies for olfactory displays—devices that actively release odor stimuli in synchronization with visual and auditory cues—are advancing rapidly. With advances in microelectromechanical system (MEMS)-based scent dispensers, microheaters, and multichannel cartridge systems, miniaturization and precise control have become increasingly feasible, expanding their applications in immersive VR/AR, telepresence, and HRI scenarios [4, 20, 21]. More recently, closed-loop odor feedback systems that integrate advanced gas sensor arrays with scent output units have been proposed; these are considered essential for reproducing realistic olfactory experiences in artificial platforms [22–24].

At the core of these developments lies the choice and design of sensing materials. The physical and chemical properties of each material—such as structure, reaction mechanism, selectivity, sensitivity, and stability—directly influence device performance and must be optimized according to the intended application. Accordingly, this review provides a comprehensive overview of recent advances in gas sensing materials, focusing on organic, inorganic, and hybrid systems, and analyzes their suitability for integration into real-time, closed-loop olfactory display platforms.

Despite notable progress, challenges remain—such as delayed sensor dynamics, odor cross-contamination, device miniaturization, and drift—that hinder the realization of immersive and safe user experiences. Addressing these barriers demands next-generation gas sensor platforms characterized by ultrafast response, high selectivity, adaptive closed-loop control, and compatibility with scalable, wearable architectures. Recent breakthroughs in machine-learning-driven sensor arrays, MEMS actuators, and bio-inspired hybrid composites highlight promising directions for olfactory interfaces that deliver robust, precise, and user-centric odor experiences. Accordingly, this review emphasizes material innovations and integrated strategies specifically tailored to overcome these bottlenecks and accelerate the development of practical olfactory display systems. While the electronic nose concept provides valuable historical and technological context, the present review focuses specifically on gas sensors as core components for olfactory display systems, highlighting how recent material innovations can meet the unique requirements of closed-loop, real-time odor delivery.

### Introduction of target sensing gases

In modern gas sensor technologies, ethylene (C₂H₄), hydrogen sulfide (H₂S), hydrogen (H₂), acetone (C₃H₆O), and nitrogen dioxide (NO₂) are major target gases across diverse applications, including the food industry, industrial safety, energy management, medical diagnostics, and environmental monitoring. The biological and chemical characteristics of these gases, along with their societal impacts, are critical factors shaping the direction of sensor development (Fig. 1b) [25–30].

In research on olfactory displays and gas sensors, the primary target gases selected for this study—ethylene, hydrogen sulfide, hydrogen, acetone, and nitrogen dioxide—hold significant importance for olfactory stimulation, user safety, and environmental or health monitoring in real-world applications. The selection criteria can be summarized in three main points. First, olfactory specificity and real sensory relevance were core considerations. For instance, ethylene can represent the scent of freshness, such as fruit ripening, while H₂S and NO₂ are crucial for signaling unpleasant odors or warnings related to food spoilage and industrial environments. Acetone, detectable in human breath, serves as a biomarker for metabolic diseases like diabetes, and although H₂ is odorless, its detection is vital for safety monitoring because of its explosive risk and future energy management. Second, technological applicability and breath of use were considered. These gases are widely studied as representative models for evaluating sensor performance—sensitivity, selectivity, and rapid response—and have broad use in real-world contexts including food, healthcare, industry, and urban environments. Third, practical reproducibility and realistic composition in olfactory display systems were considered. In complex smell environments and human–machine interaction scenarios, these gases function as key olfactory cues, warning signals, or biomarkers essential for enhancing user immersion and safety within the system.

Ethylene (C₂H₄) is a major target gas in the food industry, functioning as a critical plant hormone that regulates the ripening and spoilage of fruits and vegetables [31, 32]. Produced by most plants, this colorless gas acts as a signaling molecule that triggers major stages of growth, ripening, and the plant life cycle [33, 34]. Often referred to as the “ripening hormone,” ethylene induces biochemical transformations such as fruit softening, color change, and the development of flavor and aroma. However, excessive ethylene exposure after optimal ripeness accelerates decay and spoilage [33, 35, 36]. Fruits with high ethylene production, such as apples, bananas, and tomatoes, can stimulate the ripening of nearby produce, further accelerating spoilage [37, 38]. Consequently, ethylene accelerates the ripening process and promotes spoilage, leading to significant food loss and economic damage. Real-time monitoring of ethylene concentrations is therefore essential for extending shelf life and maintaining the quality of fresh produce during storage and distribution [39]. In addition to its biological role, ethylene is a crucial feedstock in the petrochemical industry, used for the production of polyethylene and various other plastics and chemical derivatives [40]. In industrial environments, ethylene leakage poses an explosion hazard, highlighting the need for sensitive and reliable ethylene sensors for safety management [41]. Taken together, ethylene is a vital target gas across multiple domains—including food preservation, industrial safety, and environmental monitoring—due to its diverse roles and significant impact.

Hydrogen sulfide (H₂S) is recognized as a highly toxic gas, commonly encountered in the oil and gas industry, where its detection is vital for worker safety and environmental protection [42–44]. H₂S is generated as a byproduct of various industrial processes, including natural gas and petroleum extraction, wastewater treatment, and pulp and paper manufacturing [45–47]. Even at low concentrations, H₂S poses significant health hazards, and exposure to high levels can lead to acute poisoning and potentially fatal outcomes [42, 48]. The gas directly affects the nervous system and can cause olfactory paralysis, making it difficult to detect hazardous concentrations by smell alone, which further increases the risk [49]. Given its frequent co-generation with natural gas, biogas, and petroleum, H₂S has emerged as a critical target gas requiring high-precision detection in energy production systems [50]. Consequently, H₂S sensing technologies have garnered significant attention in the field of sensor development [50, 51].

Hydrogen (H₂) is a colorless, odorless, and highly flammable gas, regarded as a promising future energy source, but also posing significant explosion risks [52–54]. Hydrogen fuel cells are recognized for their environmentally friendly and efficient energy production, making them attractive for a wide range of applications, including automobiles and power plants [55]. However, due to its wide flammability range (4–75% in air), hydrogen poses a high risk of explosion, necessitating highly sensitive and accurate leak detection systems. Hydrogen leaks during production, storage, transportation, or utilization can lead to severe fire and explosion hazard [56–58]. Although hydrogen rapidly disperses due to its low molecular weight, it can still accumulate to dangerous concentrations in confined or poorly ventilated areas. With the ongoing expansion of hydrogen-related infrastructure, such as fuel cell vehicles and hydrogen refueling stations, the demand for reliable hydrogen sensing technologies continues to grow [59]. Therefore, precise detection of hydrogen leaks throughout extraction, transportation, and utilization processes is crucial for the safe operation of rapidly developing hydrogen infrastructure [60]. Therefore, reliable hydrogen detection is indispensable for ensuring the safe operation of emerging hydrogen-based energy infrastructure.

Acetone (C₃H₆O) is a volatile organic compound (VOC) present in human breath and industrial environments, serving as a non-invasive biomarker for diabetes and a safety concern in workplaces [61–63]. Notably, elevated levels of acetone are often detected in the breath of individuals with diabetes, making it a promising non-invasive biomarker for diabetes diagnosis. Studies have shown that exhaled acetone levels are significantly higher in individuals with diabetes compared to healthy subjects, providing a foundation for breath-based diabetes monitoring [64–66]. In industrial settings, where acetone is frequently used or stored in large quantities, continuous monitoring is essential to protect worker health and prevent fire or explosion risks in the event of a leak [67–69]. Additionally, in certain medical conditions—particularly diabetes—elevated ketone levels in the bloodstream may lead to a distinct acetone odor in the breath. This highlights the potential of breath acetone analysis as a tool for disease diagnosis and monitoring in the medical field [70, 71]. Accordingly, acetone sensing is not only essential for industrial safety but also holds significant promise for non-invasive diagnostics of metabolic disorders such as diabetes.

Nitrogen dioxide (NO₂) is a reddish-brown gas with a pungent odor, identified as a significant air pollutant and a major target for environmental monitoring and public health protection. NO₂ is primarily emitted into the atmosphere through the combustion of fossil fuels in vehicles, buses, trucks, and industrial facilities. Owing to its strong oxidizing nature, NO₂ readily reacts with flammable and reducing substances, leading to the formation of nitric acid and other nitrogen oxides [72–74]. NO₂ plays a major role in the formation of photochemical smog, acid rain, and tropospheric ozone, all of which pose serious environmental and health risks [75]. Vulnerable populations, including children, the elderly, and individuals with pre-existing respiratory conditions, are particularly sensitive to elevated NO₂ concentrations. Therefore, NO₂ sensors are essential for air quality monitoring in urban areas and serve as a crucial component of air pollution alert systems [76, 77]. Thus, NO₂ remains a critical target for next-generation gas sensors, both as a key pollutant in urban air quality monitoring and as a safeguard for public health.

These five gases play critical roles across a wide range of essential sectors in modern society, making them major targets for the development of advanced gas sensor technologies. Ethylene requires precise monitoring for quality control and waste reduction in the food industry; hydrogen sulfide for industrial safety and worker protection; hydrogen for ensuring the safety of next-generation energy infrastructure; acetone for advancing medical diagnostics; and nitrogen dioxide for environmental protection and public health. The diverse, application-specific demands associated with these gases continue to drive the advancement of gas sensing technologies. Accordingly, the development of tailored sensors that address the unique properties and risks associated with each gas remains an active area of research. Accordingly, these gases were prioritized not only as representative analytes in sensing research but also as functional cues and safeguards in olfactory display pipelines—ranging from freshness representation (ethylene) to safety alerts (hydrogen sulfide, hydrogen, nitrogen dioxide) and non-invasive health monitoring (acetone), thereby bridging sensing science with immersive olfactory technologies.

## Organic Materials-based gas sensors

Organic material-based gas sensors are being actively researched as next-generation gas sensor platforms. This is due to their excellent flexibility, lightweight nature, low-temperature processability, and potential for mass production through solution-based processing. They are garnering significant attention for their ability to detect various gases, including volatile organic compounds (VOCs), toxic gases, and biogases, with high sensitivity and selectivity in real time. Organic material-based gas sensors detect gases by generating electrical, optical, or mass changes through selective chemical or physical interactions with specific gas molecules [78, 79]. Major organic materials utilized as the sensor’s active layer include conductive polymers, organic semiconductors, biomolecules (e.g., proteins, DNA, enzymes), and fluorescent organic molecules. For instance, conductive polymers undergo significant changes in conductivity through redox reactions or doping/de-doping processes involving gas molecules. Organic semiconductors, on the other hand, regulate current flow due to changes in their energy band structure upon gas adsorption. Biomolecules leverage highly specific enzymes, antibodies, and DNA to convert biochemical reactions with particular gas molecules into electrical or optical signals, enabling the selective detection of trace amounts of gas. These organic materials exhibit reversible changes in electrical conductivity, luminescence properties, or color in response to interactions with gas molecules [80–82]. They can be composed of various material components (e.g., organic/organic and organic/inorganic combinations) [83]. Together, these features highlight the versatility of organic materials as functional building blocks for flexible, selective, and wearable gas sensing devices in olfactory display applications.

### Composite organic materials-based gas sensors

**Fig. 2:**
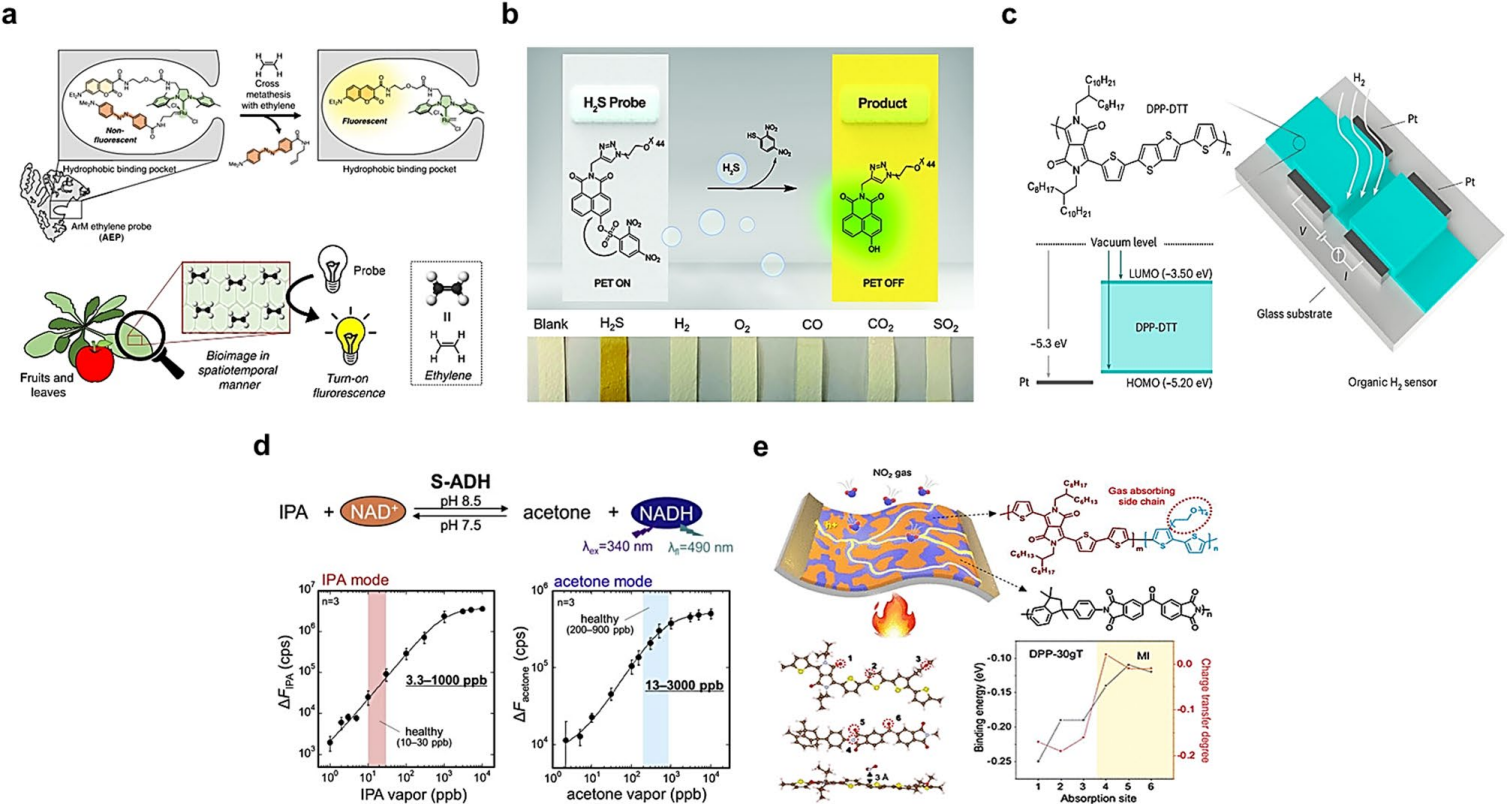
Representative sensing mechanisms and structural illustrations of organic material–based gas sensors. **a** Ethylene sensing mechanism of an albumin-stabilized ruthenium metalloenzyme biosensor, where ethylene triggers a cross-metathesis reaction resulting in fluorescence activation and electrochemical signal changes. Reproduced with permission from [84]. Copyright 2019, Springer Nature. **b** H₂S detection using an azide-functionalized fluorescent probe, which undergoes rapid azide-to-amine reduction with a visible fluorescence turn-on. Reproduced with permission from [85]. Copyright 2021, Royal Society of Chemistry. **c** H₂ sensing via an organic thin-film transistor (OTFT) employing DPP-DTT as the active layer, enabling reversible p-doping/de-doping behavior. Reproduced with permission from [86]. Copyright 2025, Springer Nature. **d** Acetone detection using an enzymatic fiber-optic flow cell with S-ADH and NAD⁺/NADH system for real-time monitoring. Reproduced with permission from [87]. Copyright 2021, Elsevier. **e** NO₂ sensing by a conjugated polymer blend sensor with polar side chains, achieving ppb-level chemiresistive response. Reproduced with permission from [88]. Copyright 2023, American Chemical Society. These examples demonstrate the diversity of organic material platforms (proteins, polymers, enzymes, and fluorophores) for selective, real-time, and room-temperature gas sensing toward olfactory display applications

Organic gas sensing materials, which lack inorganic or hybrid components, are primarily composed of conjugated polymers, small organic molecules, proteins, peptides, and nucleic acids. These materials exhibit chemiresistive, optical, or fluorescence-based sensing mechanisms, depending on their molecular structure and device integration. Their intrinsic flexibility, low-temperature processability, and tunable chemical functionality make them attractive for wearable and disposable artificial olfaction systems, especially for applications requiring lightweight, biocompatible, and highly selective sensing under ambient conditions.

Recent studies have reported protein-, peptide-, and phage-based ethylene sensors. These platforms offer high selectivity and real-time optical readout, which is useful for olfactory display feedback. As shown in Fig. 2a, an artificial metalloenzyme biosensor was developed using albumin to stabilize a ruthenium-based catalyst in an aqueous matrix. Ethylene triggers a cross-metathesis reaction with the catalyst, releasing a quencher and activating fluorescence. The biosensor also exhibits electrochemical signal changes upon ethylene binding. The protein scaffold enhances catalyst stability against biological deactivation. This system enables real-time ethylene detection from biological sources at ppm-level sensitivity [84]. A colorimetric ethylene sensor was developed using thiol-modified polydiacetylene (PDA), which changes color from blue to red upon ethylene exposure. The PDA backbone responds to structural changes induced by thiol–ethylene interactions, allowing visual detection without complex equipment. Its compatibility with flexible substrates allows various device formats. The sensor demonstrated a detection limit of 600 ppm under ambient conditions with high ethylene selectivity [89]. High-mobility polymer nanofibrils based on DPPT-TT and P(NDI2OD-T2) were used in OFETs for ethylene sensing. The nanofibrils create efficient charge pathways and respond to gas adsorption by modulating electrical signals. The device showed high sensitivity and multi-gas detection capabilities. This approach is particularly applicable to agriculture and food quality monitoring for ethylene detection [90]. A hydrogen-bonded organic framework using tetracyano-bicarbazole enables selective ethylene/ethane separation via size-specific micropores and strong intermolecular interactions. The material exhibits a gating mechanism, increasing ethylene adsorption above a threshold pressure. While primarily designed for gas separation, the HOF’s high ethylene selectivity suggests strong potential for use in ethylene gas sensors [91].

Various organic material-based H₂S gas sensors have been developed with enhanced sensitivity and selectivity by employing reaction-based fluorescent probes and peptide-functionalized films. A color turn-on fluorescent probe based on a DDM (azide-functionalized fluorophore) structure enables real-time H₂S detection and food spoilage monitoring as shown in Fig. 2b. The probe utilizes an azide-to-amine reduction mechanism, exhibiting a detection limit of 58 nM, a rapid fluorescence response within 30 s, and clear visible color change for practical food safety applications [85]. A genetically encoded fluorescent sensor using p-azidophenylalanine enables selective and reversible detection of H₂S in live cells. The reaction with H₂S induces conformational and fluorescence changes in the protein, allowing submicromolar detection via FRET. This system successfully tracks real-time endogenous H₂S production, offering high spatial and temporal resolution [92]. Mito-NPNM and Lyso-NPNM are N-annulated perylene-based fluorescent probes for selective H₂S detection in mitochondria and lysosomes. They rely on nitro-to-amine reduction, yielding dual-emission fluorescence and a visible color change. A large spectral shift (~ 200 nm) supports ratiometric detection with 139 nM sensitivity in biological environments, including serum and live cells [93]. A naphthalimide-based probe detects H₂S via Michael addition-triggered intramolecular cyclization, yielding a 75-fold fluorescence increase. It shows strong selectivity over other thiols and a detection limit of 0.23 µM. The probe supports real-time intracellular imaging and rapid H₂S detection (< 5 min), highlighting its biomedical applicability [94].

Various organic hydrogen gas sensors have been developed using conductive polymers like polyaniline and chitosan-based composites, enabling ppm-level detection at room temperature. An organic hydrogen gas sensor using DPP-DTT as the active layer exploits the reversible p-doping/de-doping behavior induced by oxygen in the presence of H₂ as illustrated in Fig. 2c. Integrated into an OTFT structure, the device exhibits high charge mobility, enabling rapid and reversible threshold voltage shifts. The sensor demonstrates a fast response time of 0.84 s, a detection limit of 192 ppb, and excellent cycle stability, supporting its potential for real-time H₂ monitoring [86]. A hydrogen gas sensor utilizing nanostructured polyaniline with a 3D porous architecture achieves high sensitivity through increased surface area and enhanced interaction with hydrogen. The sensor operates at room temperature, detecting H₂ down to 1 ppm with 29% sensitivity and response/recovery times of 15/17 s. It also exhibits exceptional long-term stability (646 days) and ultra-low power consumption (< 2 µW), making it ideal for low-power sensing networks [95]. A room-temperature electrochemical H₂ sensor was developed using a chitosan/PANI composite hydrogel with a 3D porous structure. The hydrogel enhances gas adsorption and charge transport while enabling impedance-based H₂ detection over a wide range (0.15–1,500 ppm). The sensor exhibits a fast response time of 1.9 s and stable performance under ambient conditions, demonstrating its potential for practical applications [96].

Various organic material-based acetone gas sensors have been developed with enhanced selectivity by designing fluorescent probes and peptide-functionalized polymer films for breath detection. The sensor utilizes a fiber-optic flow cell with immobilized S-ADH and the NAD⁺/NADH enzyme system to detect acetone and isopropanol in breath in real time. Acetone is enzymatically converted to isopropanol, resulting in changes in NADH concentration that are measured via fluorescence as illustrated in Fig. 2d. By altering the pH and enzyme solution, both gases can be detected. It operates reliably within a range of 13–3,000 ppb and is suitable for non-invasive monitoring of lipid metabolism [87]. This sensor features a multilayer Nafion/enzyme-coated electrode, where S-ADH catalyzes the reduction of acetone to iso-propanol, coupled with NADH oxidation. The subsequent cascade reaction produces hydrogen peroxide, the oxidation current of which is measured amperometrically to quantify acetone. It offers a wide detection range from 1 µM to 25 mM in liquid and ppm levels in gas, with a detection limit of 0.03 µM (approximately 1.7 ppb). Its solid electrolyte design prevents evaporation and leakage, making it suitable for portable diabetes and ketosis monitoring [97].

Various organic material-based NO₂ gas sensors have been developed by using conjugated polymers and peptide films to achieve oxidation-responsive electrical or optical signal changes. As shown in Fig. 2e, a thermally stable chemiresistive NO₂ sensor was developed using conjugated polymer blends with polar side chains (e.g., DPP-based polymers with oligoethylene glycol side chains. The sensor enables real-time monitoring by exhibiting resistance changes upon NO₂ exposure, with a detection limit of 100 ppb and rapid response/recovery times of 15/30 s [88]. The triazine-core 2D conjugated polymer (T-2DP) nanosheet sensor features nanopores and electron-withdrawing –CN/N sites that facilitate strong π–π stacking and charge-transfer interactions with NO₂. It achieves high sensitivity (452.6 ppm⁻¹), a detection limit below 100 ppb, and response/recovery times of ~ 35/47 s. Its performance on flexible substrates highlights its potential for wearable gas sensing [98]. The 3D multilayer P3HT channel sensor with micro–mesopores enhances gas diffusion and adsorption area while relieving mechanical strain. It exhibits a response increase from 122 to 1,053% at 30 ppm NO₂, a low detection limit (approximately 2.3 ppb), and quick response/recovery times of ~ 10/20 s. It maintains performance under a 1 mm bending radius, demonstrating high flexibility [99]. Collectively, these examples illustrate the breadth of organic sensing strategies—from fluorescence probes to enzyme-based systems—and underscore their potential for achieving ultralow detection limits and high selectivity, while also emphasizing the need for greater stability and compact integration.

As summarized in Table 1, recent developments in organic material–based gas sensors reveal distinct strengths and trade-offs depending on the sensing modality and device architecture. Fluorescence- and colorimetric-based sensors, often employing protein or enzyme systems, deliver ultralow detection limits—reaching the nanomolar to sub-ppb range for analytes such as H₂S and acetone—through highly specific biological recognition. This exceptional selectivity is advantageous for applications such as medical breath diagnostics and food spoilage monitoring. However, the requirement for bulky optical readout components and the susceptibility to ambient humidity and biofouling hinder their integration into compact, real-time, or wearable olfactory display platforms. In contrast, electrical transduction methods, including organic field-effect transistor (OFET)–based sensors using semiconductors such as poly{[N, N′-bis(2-octyldodecyl)-naphthalene-1,4,5,8-bis(dicarboximide)-2,6-diyl]-alt-5,5′-(2,2′-bithiophene)} (DPP-DTT) achieve sub-ppb hydrogen detection with response and recovery times often within only a few seconds, making them highly promising for real-time gas monitoring and seamless system-level integration. These platforms excel in miniaturization potential and compatibility with digital electronics, though their selectivity toward specific odorants can be lower than that of enzyme-based systems. Mitigating cross-sensitivity and long-term drift through advanced material engineering remains an ongoing focus. Hybrid and composite approaches, such as functionalized polymer films or enzyme-embedded membranes, provide a balance between tunable selectivity, mechanical flexibility, and robust device integration. Nonetheless, improvements in the operational stability of biofunctional layers and reproducibility under varying environmental conditions are still needed. Overall, organic material–based sensors offer inherent advantages in flexibility, functional tunability, and biocompatibility over conventional inorganic systems. However, achieving long-term stability, environmental tolerance, and compact integration will be critical for their deployment in practical, user-centric olfactory display applications.

**Table 1:** Sensing performance of various organic materials-based gas sensors

Sensing Gas	Sensing Materials	Detection Method/Technology	Working Temperature (℃)	Concentration (ppm)	Response Time (s)	Recovery Time (s)	Reference
Ethylene	Albumin–Ru	Optical	25–37	27	< 60	-	[84]
	Polydiacetylene	Optical	Room Temp.	200	< 480	-	[89]
	Mycobacterium	Optical	Room Temp.	0.009	-	-	[90]
	Tetracyano-bicarbazole	Optical	333 K (60)	-	-	-	[91]
H_2_S	Polymer composite	Optical	Room Temp.	0.09 µM	10	< 60	[85]
	Azide‑substituted GFP	Optical	25–37	nM value	< 30	-	[92]
	N‑annulated perylene	Optical	Room Temp.	0.1–3.5 µM	< 30	< 60	[93]
	Michael-acceptor	Optical	Room Temp.	0.23 µM	< 60	-	[94]
H_2_	DPP‑DTT	Electrical	Room Temp.	0.192	< 1	-	[86]
	PANi	Electrical	Room Temp.	1	< 15	17	[95]
	Chitosan/polyaniline	Electrochemical	Room Temp.	0.15–1,500	< 1.9	< 2	[96]
Acetone	S‑ADH/Nafion	Electrical	Room Temp.	0.013	< 10	< 10	[87]
	Nafion	Electrochemical	Room Temp.	0.002	< 30	-	[97]
NO_2_	Polymer	Optical	Room Temp.	130	< 15	< 30	[88]
	Triazine	Electrical	Room Temp.	0.1	< 35	< 47	[98]
	Polymercomposite	Optical	Room Temp.	0.002	< 10	< 20	[99]

## Inorganic materials-based gas sensors

Inorganic materials form the cornerstone of traditional gas sensor technologies due to their high thermal stability, robustness, and reliable sensing performance under harsh environmental conditions. These materials, particularly MOS, have been extensively investigated over the past few decades [100, 101] and remain central to both commercial and research-level artificial olfaction systems. Inorganic sensors operate predominantly via chemiresistive mechanisms, in which gas adsorption on the sensor surface induces changes in electrical resistance and are capable of detecting a wide range of oxidizing and reducing gases. Recent advances in nanostructuring, doping, and morphology control have greatly enhanced their response characteristics [17, 102, 103], making them strong candidates for integration into next-generation olfactory platforms. In this section, we provide an overview of gas sensors based on both pristine and composite inorganic materials.

### Pristine inorganic materials-based gas sensors

**Fig. 3:**
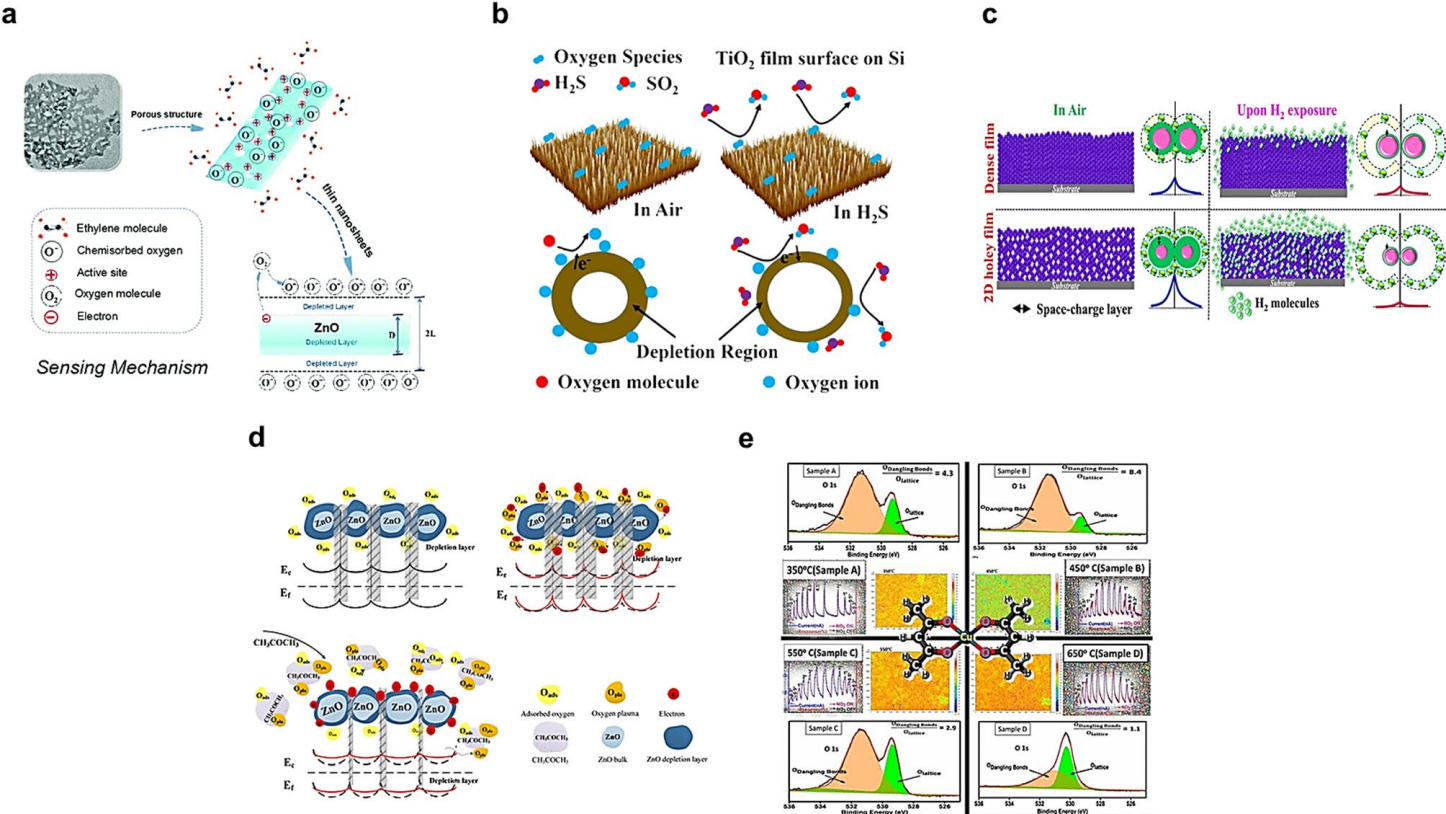
Representative sensing mechanisms and structural illustrations of pristine inorganic gas sensors. **a** Ethylene detection using porous ZnO nanosheets, where adsorbed oxygen ions react with ethylene to release conduction electrons. Reproduced with permission from [104]. Copyright 2019, Royal Society of Chemistry. **b** H₂S sensing by TiO₂ thin films, relying on surface chemisorption and electron transfer. Reproduced with permission from [107]. Copyright 2021, Elsevier. **c** H_2_ detection using unholey/holey 2D ZnO sensors that undergo a semiconductor-to-metal transition upon exposure. Reproduced with permission from [112]. Copyright 2021, Elsevier. **d** Acetone detection with ZnO nanofibers, showing modulation of the depletion layer thickness and surface potential barriers. Reproduced with permission from [117]. Copyright 2020, American Chemical Society. **e** NO₂ detection with CuO nanostructures, where NO₂ adsorption increases hole concentration and decreases resistance. Reproduced with permission from [126]. Copyright 2021, American Chemical Society. Together, these inorganic platforms highlight the robustness, thermal stability, and surface chemistry-driven selectivity of pristine materials for integration into next-generation olfactory display systems

As summarized in Table 2, pristine inorganic gas sensing materials—which lack polymeric or hybrid components—are primarily composed of MOS, two-dimensional inorganic compounds, and certain ceramic materials. These materials exhibit chemiresistive, electrochemical, or capacitive sensing mechanisms, depending on their composition and device configuration. Their inherent high thermal stability, chemical durability, and structural simplicity make them attractive for long-term use in artificial olfaction systems, especially for applications requiring stability under fluctuating environmental conditions.

As shown in Fig. 3a, porous ZnO nanosheets operate via a chemiresistive mechanism [104]. Adsorbed oxygen (O⁻, O₂⁻) traps conduction electrons; ethylene reverses this process and lowers resistance. Upon exposure to ethylene, these ions react and release electrons back into the ZnO conduction band, reducing the resistance. The ultrathin, high-surface-area porous architecture promotes rapid gas diffusion and electron transfer. This sensor exhibits a detection limit as low as 10 ppm, with a response/recovery times of ~ 8/20 s, operating optimally at 500 °C, achieving faster response dynamics than conventional MOS sensors. A LaFeO₃-based perovskite gas sensor [105] demonstrated high selectivity to ethylene and acetylene by tuning the operating temperature. The sensing mechanism involves changes in electrical resistance via oxygen vacancy-mediated adsorption/desorption of ethylene. The LaFeO₃-based sensor exhibited response and recovery times of approximately 45/75 s, respectively, at 150 °C, demonstrating strong and reversible signals with excellent thermal and humidity stability. Recently, a chemiresistive ethylene sensor based on sheet-like hierarchical Co₃O₄ structures [106] exhibited enhanced sensing performance due to its porous morphology and high surface defect density. The Co₃O₄ 6 h variant, characterized by abundant oxygen vacancies, demonstrated a high response of 49.6 to 100 ppm ethylene at 100 °C, with response/recovery times of 27/42 s, respectively. DFT calculations confirmed that ethylene detection occurs via physisorption-driven electron transfer processes, with performance governed by surface oxygen vacancy density and adsorption energetics.

Various metal oxide-based H₂S gas sensors have been developed with enhanced sensitivity and selectivity by tailoring their nanostructures and surface properties. As shown in Fig. 3b, TiO₂ thin film sensors [107] operate based on chemisorbed oxygen species that react with H₂S to release electrons, thereby modulating the film’s conductivity. Their performance is enhanced by high surface roughness and nanocrystallinity, enabling fast response (~ 15 s) within an operating temperature range of 200–350 °C. In₂O₃ colloidal quantum dot sensors [108] function near room temperature through surface charge modulation upon H₂S exposure, made possible by ligand exchange and partial ligand removal to expose active sites. These sensors demonstrate rapid response/recovery with detection at low ppm levels, offering promise for low-power, portable applications. NiO-based porous microsphere sensors [109] leverage oxygen species interaction and the catalytic activity of Ni sites, showing excellent reversibility with a detection limit of 0.25 ppm, rapid response/recovery times of 5/10 s at an operating temperature of 200 °C. SnO₂ porous nanofiber sensors [110], fabricated via electrospinning, utilize surface depletion layer modulation and grain boundary control to achieve high sensitivity. Operating at 350 °C, they exhibit an ultra-low detection limit of 1.6 ppb, with a response/recovery times of 15/230 s. Lastly, pristine α-Fe₂O₃ nanofiber sensors [111] prepared by on-chip electrospinning operate via chemiresistive mechanisms involving nanograin homojunctions and oxygen species reactions. Without any dopants or additives, the optimized sensor achieved a response of ~ 6.1 to 1 ppm H₂S at 600 °C, demonstrating stable detection capability, although the response/recovery times were not explicitly reported.

H_2_ gas detection has garnered considerable attention due to its critical role in safety monitoring, with metal oxide–based sensors [114, 116] offering high sensitivity and rapid response characteristics. As shown in Fig. 3c, a room-temperature H₂ sensor utilizing holey-engineered 2D ZnO nanosheets [112] enhances gas diffusion and reactive surface area through in-plane nanopores. The sensing mechanism is based on a semiconductor-to-metal transition: hydrogen reduces ZnO to Zn, thereby releasing electrons back into the conduction band and sharply decreasing the resistance. The optimized ZnO@400°C sample shows a detection limit of 100 ppm, a fast response/recovery times of 9/6 s, and excellent repeatability (∼97–99% over 45 days), making it a promising platform for real-time hydrogen leak detection. SnO₂ thin films [118], fabricated via magnetron sputtering, utilizes surface crystal orientation to enhance gas selectivity and response characteristics. The sensing mechanism is governed by the modulation of surface oxygen species: oxygen molecules adsorbed on specific SnO₂ crystal facets form O⁻ or O₂⁻ ions by capturing electrons from the conduction band. Upon exposure to H₂, these ions react with hydrogen to form water, releasing trapped electrons back into the conduction band, and thereby reducing the resistance of the n-type semiconductor. The sensor exhibits a detection limit of approximately 100 ppm, a fast response time (~ 19 s at 250 °C), and high selectivity, with improved performance attributed to the optimized density of active sites and oxygen vacancies on exposed high-energy facets. Another material for hydrogen gas sensors is CuO. This sensor, based on nano-patterned polycrystalline CuO nanowires integrated into a nanogap electrode (~ 33 nm), exploits void-induced enhancement in gas diffusion and electric field-assisted charge modulation [119]. The voids, formed by controlled pre-annealing and oxidation, significantly increase surface reactivity. The sensor demonstrates a record-low detection limit of 5 ppb, response/recovery times of 5/10 s, respectively, under a bias-induced electric field (~ 1.3 MV cm⁻¹), and robust stability under 50% relative humidity without Cu(OH)₂ formation. Its excellent repeatability and fast kinetics make it a strong candidate for real-time H₂ monitoring applications.

Various MOS have been widely investigated for acetone gas detection due to their tunable surface chemistry and stable chemiresistive behavior under thermal activation. ZnO nanofiber-based sensors fabricated via electrospinning and oxygen plasma treatment exhibit enhanced acetone-sensing performance through modulation of surface oxygen vacancies and charge carrier transport as shown in Fig. 3d. The plasma treatment significantly increases the density of active sites and reduces the conduction barrier, leading to improved response characteristics. The sensor demonstrates optimal performance at 250 °C, with a response/recovery times of 9/10 s, providing strong signal reproducibility and stability [117]. In the case of Co₃O₄, hierarchical one-dimensional nanofibers constructed through a template-free hydrothermal process offer a high surface area and efficient charge transfer pathways. As a p-type semiconductor, Co₃O₄ exhibits resistance decrease upon acetone exposure, with excellent performance at a low operating temperature of 190 °C, and ultrafast dynamics with response/recovery times of 7/1 s, respectively [120]. Fe₂O₃-based acetone sensors, synthesized in foam-like porous structures, leverage enhanced gas diffusion and increased adsorption sites due to the high porosity. The sensor operates at 300 °C, exhibiting measurable response at sub-ppm acetone concentrations; however, specific response/recovery times were not explicitly reported [121]. CuO thin films deposited on flexible Kapton substrates have also been utilized for wearable breath sensors. As a p-type oxide, CuO undergoes resistance decrease upon exposure to reducing gases like acetone. Its porous microstructure and good film-substrate adhesion enable stable performance at 150 °C, and while precise dynamic response values were not stated, its rapid signal shifts in exhaled breath detection highlight its sensitivity [122]. Lastly, SnO₂ nanosheet-based sensors with a loose lateral morphology provide high surface area and gas permeability. As an n-type semiconductor, SnO₂ responds to acetone by decreasing resistance due to the reaction between pre-adsorbed oxygen ions and acetone molecules. The sensor exhibited excellent performance at 250 °C, with an ultrafast response/recovery times of 2/3 s, along with a detection limit in the ppt-level range [123].

Indium oxide (In₂O₃), a well-known n-type semiconductor, has shown excellent performance for NO₂ detection due to its high surface reactivity and abundant oxygen adsorption sites. Nanoparticle-packed In₂O₃ nanospheres synthesized via a solvothermal route exhibit high sensitivity, achieving a response of 217.5 to 500 ppb NO₂, with a detection limit below 10 ppb [124]. The sensing mechanism is governed by electron depletion upon interaction with oxidizing NO₂ molecules, which extract electrons from the conduction band and modulate surface resistance. These sensors operate optimally at 120 °C, demonstrating fast and reversible response behavior suitable for low-concentration NO₂ monitoring. Similarly, mesoporous polycrystalline SnO₂ frameworks synthesized using a soft templating method offer a large specific surface area and open porous pathways [125], enhancing gas accessibility and diffusion. These characteristics facilitate efficient electron exchange at the gas–solid interface, leading to a response/recovery times of 90/175 s, and strong NO₂ sensitivity at an operating temperature of 150 °C. ZnO nanoflowers, formed via hydrothermal growth, present radially aligned nanorods with exposed (0001) facets that act as active sites for NO₂ adsorption [114]. As an n-type semiconductor, ZnO responds to NO₂ via electron capture, increasing the resistance of the sensing layer. This sensor exhibits a response of ~ 22.8 to 5 ppm NO₂ at room temperature, with response/recovery times of 130/190 s, respectively. The flower-like morphology contributes to enhanced surface interaction and gas diffusion efficiency. WO₃ nanofibers prepared with engineered porosity and oxygen vacancy control (especially those annealed at 10 °C/min) display enhanced low-temperature NO₂ sensing performance [116]. These nanostructures, owing to their mesoporous framework and oxygen vacancy-induced activation sites, achieve a high response of 101.3 to 5 ppm NO₂ at 90 °C, with a response/recovery times of 125/231 s, making them promising for sensitive and low-power gas detection. Finally, pristine CuO nanoflakes, a p-type semiconductor, operate through a different mechanism, as shown in Fig. 3e, in which NO₂ molecules, acting as electron acceptors, withdraw electrons from adsorbed oxygen species on the CuO surface [126], thereby increasing hole concentration and decreasing resistance. Without any conductive additives such as reduced graphene oxide, the pristine CuO sensor demonstrates a moderate response of ~ 82% to 5 ppm NO₂ at room temperature (23 °C), with slightly slower dynamics than its rGO-composite counterpart. Although the response/recovery times are not explicitly reported, the bare CuO sensor shows stable operation and reasonable reversibility, highlighting its viability for ambient-temperature NO₂ sensing. The sensor also demonstrated low hysteresis (< 20%) and high stability over a two-week testing period, highlighting its strong potential for practical, low-power gas detection applications. Taken together, these results confirm that pristine inorganic semiconductors provide robust thermal stability and high sensitivity, though their reliance on elevated operating temperatures continues to limit their practical integration into wearable olfactory systems.

**Table 2:** Sensing performance of various pristine inorganic materials-based gas sensors

Sensing Gas	Sensing Materials	Detection Method/Technology	Working Temperature (℃)	Concentration (ppm)	Response Time (s)	Recovery Time (s)	Reference
Ethylene	ZnO	Chemiresistive	500	10	8	20	[104]
	LaFeO_3_	Chemiresistive	150–200	100	45	75	[105]
	Co_3_O_4_	Chemiresistive	100	100	27	42	[106]
H_2_S	TiO_2_	Chemiresistive	200–350	50	48	300	[107]
	In_2_O_3_	Chemiresistive	Room Temp.	5	72	200	[108]
	NiO	Chemiresistive	200	0.25	5	10	[109]
	SnO_2_	Chemiresistive	350	1	15	230	[110]
	α-Fe_2_O_3_	Chemiresistive	250	1	-	-	[111]
H_2_	ZnO	Chemiresistive	Room Temp.	100	9	6	[112]
	SnO_2_	Chemiresistive	120	0–2,000	65	176	[113]
	NiO	Chemiresistive	250	200	56	21	[114]
	CuO	Chemiresistive	Room Temp.	5 (ppb)	5	10	[115]
	TiO_2_	Chemiresistive	Room Temp.	500	43.0	11	[116]
Acetone	ZnO	Chemiresistive	250	100	65	75	[117]
	Co_3_O_4_	Chemiresistive	100	1	27	56	[118]
	Fe_2_O_3_	Chemiresistive	300	0.5	4	10	[119]
	CuO	Chemiresistive	150	0.05	30	45	[120]
	SnO_2_	Chemiresistive	250	5	2	3	[121]
NO_2_	In_2_O_3_	Chemiresistive	120	1	148	72	[122]
	SnO_2_	Chemiresistive	200	8	< 300	> 300	[123]
	ZnO	Chemiresistive	Room Temp.	1	360	240	[124]
	WO_3_	Chemiresistive	90	3	125	231	[125]
	CuO	Chemiresistive	Room Temp.	5	58.4	92.7	[126]

### Composite inorganic materials-based gas sensors

**Fig. 4:**
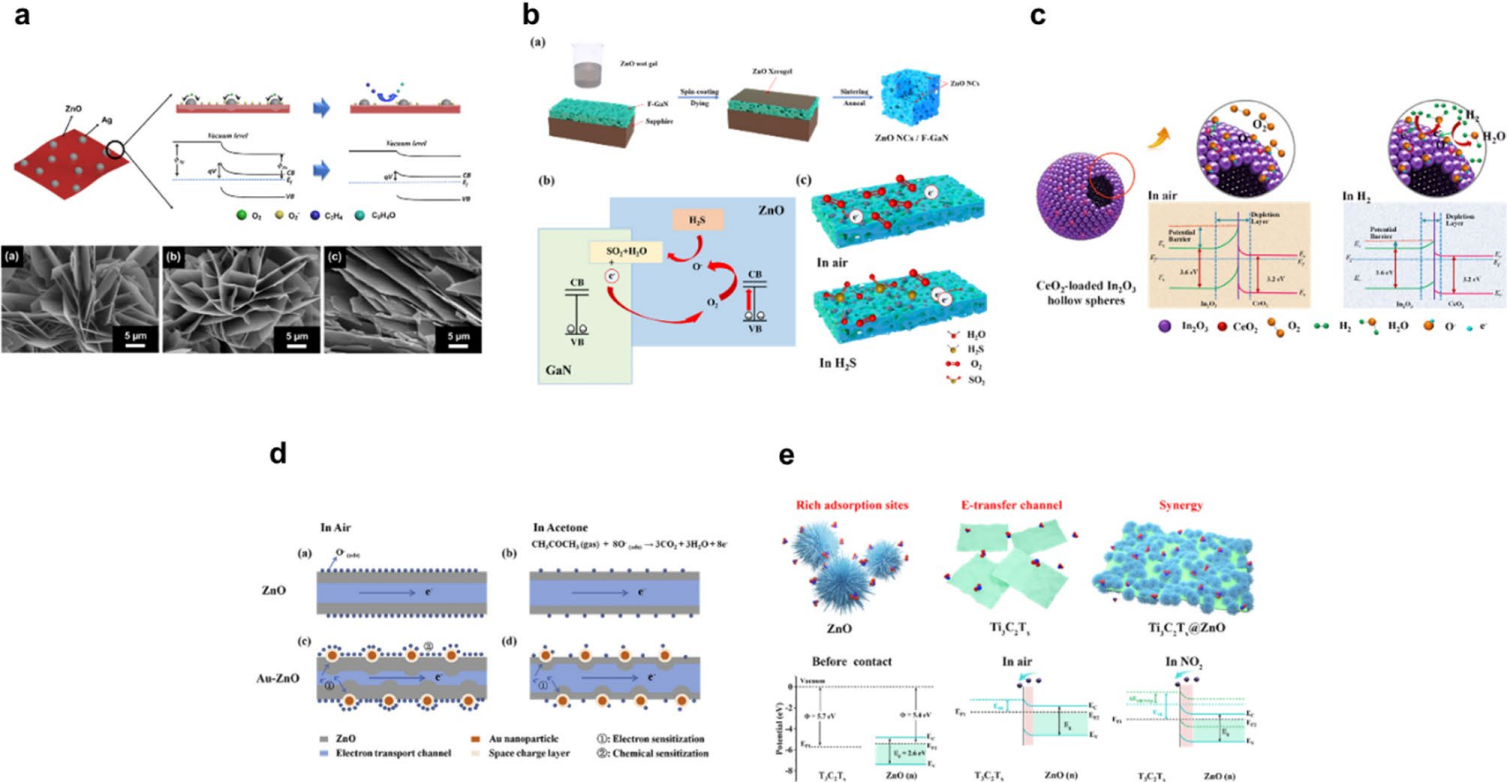
Representative sensing mechanisms and structural illustrations of various inorganic-composite gas sensors. **a** Ethylene sensing mechanism by Ag loaded ZnO and SEM images of Ag@ZnO by different Ag loading rates. Reproduced with permission from [130]. Copyright 2021, Elsevier. **b** Schematic illustration of fabrication process of the ZnO NCs@F-GaN composite nanostructure and detection/multiphase catalytic mechanism of gas sensor in air and H_2_S. Reproduced with permission from [137]. Copyright 2023, Elsevier. **c** Schematic illustration of electron transport and corresponding energy band diagrams for pure CeO₂-loaded In₂O₃ hollow spheres under air and hydrogen gas environments. Reproduced with permission from [139]. Copyright 2018, Elsevier. **d** Gas sensing mechanism of Au-ZnO under air and acetone gas environments. Reproduced with permission from [145]. Copyright 2019, Elsevier. **e** Potential gas sensing regime of the Ti_3_C_2_T_x_@ZnO composites and the energy band diagrams of the heterostructure of Ti_3_C_2_T_x_ @ZnO under various conditions. Reproduced with permission from [152]. Copyright 2023, Elsevier

Inorganic gas sensors demonstrate excellent sensitivity as well as superior thermal stability, chemical robustness, and mechanical strength, enabling reliable and durable operation even under extreme environmental conditions. Nevertheless, they often require high operating temperatures and sometimes suffer from limited gas selectivity [127]. To address these challenges, inorganic composite materials have emerged as promising candidates, combining the structural advantages of inorganic phases with enhanced interfacial properties. The integration of distinct inorganic components at the nanoscale can synergistically optimize gas adsorption behaviors, electronic structures, and surface reaction dynamics, resulting in significantly improved selectivity, sensitivity, working temperature and stability for olfactory gas sensing [128, 129]. In this section, recent advances in inorganic composite-based olfactory gas sensors are discussed, focusing on material design strategies, sensing mechanisms, and performance improvements achieved through composite engineering. Representative studies are categorized and reviewed based on the target gas species to highlight gas-specific sensing behavior and material–gas interactions. For clarity and comparison, the key performance metrics of all inorganic composite-based olfactory gas sensors discussed in this section are summarized in Table 3.

Ethylene detectable metal oxide-based sensors typically require high operating temperatures above 250 °C and exhibit slow response characteristics for its detection [133, 154]. To address these limitations, various approaches have been explored to enhance surface reactivity, including the incorporation of noble metal catalysts, metal oxide for improving surface adsorption [155] and structural modifications [154, 132]. Interestingly, this inherent low reactivity has also been exploited in an alternative strategy: by selectively oxidizing and filtering out more reactive interfering gases prior to detection, sensors can allow only ethylene to reach the active layer, thereby improving both selectivity and sensitivity. A representative example of an ethylene gas sensor by filtering process is a bilayer structure in which a thin Cr₂O₃ catalytic layer is coated onto hollow spherical SnO₂ particles [156]. The Cr₂O₃ layer selectively oxidizes and removes more reactive interfering gases due to its strong oxidative power, thereby enabling the underlying SnO₂ layer to respond specifically to ethylene. As a result, the sensor exhibited a fast response time of less than 6 s to 2.5 ppm ethylene at 350 °C, and effectively detected ethylene released during the ripening process of fruits such as bananas and blueberries. While the Cr₂O₃@SnO₂ sensor enhances ethylene selectivity by selectively oxidizing and filtering out interfering gases, the Pd-doped SnO₂ sensor improves sensitivity, response speed, and detection limit by promoting surface oxygen activation and electron redistribution [133]. Pd facilitates oxygen ion dissociation and electron trapping, directly enhancing the sensing reaction. Consequently, the Pd-doped sensor operated at a lower temperature of 250 °C, showing a response time under 1 s to 100 ppm ethylene and a detection limit of 50 ppb, offering a distinct alternative to the high-temperature Cr₂O₃-based approach. Recently, room-temperature, low-power ethylene sensors have gained increasing attention. As shown in Fig. 4a, a sensor based on electrochemically deposited ZnO–Ag nanosheets on a flexible PET-ITO substrate exhibited a maximum response of 16.01% to 100 ppm ethylene, with stable and repeatable performance [130]. Under ambient conditions, the electron-accepting and catalytic properties of Ag promoted oxygen ion adsorption and electron redistribution, enabling effective sensing without the need for high operating temperatures. However, the response/recovery times remain relatively slow—on the order of several minutes—highlighting the need for further optimization for real-time applications.

Among reducing gases, hydrogen sulfide (H₂S) is particularly toxic and chemically reactive, necessitating detection at ppb levels [157]. Similar to other reducing gases, it donates electrons and decreases the resistance of metal oxide sensors [141–148]; however, it also undergoes sulfuration reactions on the sensor surface, resulting in a more complex sensing mechanism. Consequently, achieving high sensitivity along with material and structural designs that prevent irreversible damage from sulfide formation is critical in H₂S sensor development [158]. A representative H₂S gas sensor involves a composite structure combining ZnO nanocrystals with foam-like GaN (F-GaN), as illustrated in Fig. 4b [137]. The porous F-GaN framework facilitates the adsorption of oxygen anions and promotes stepwise catalytic reactions through enhanced electron transfer and oxygen activation, thereby improving sensing efficiency. As a result, the sensor exhibited excellent performance, achieving a response of 89.7 to 50 ppm H₂S at 220 °C, with response/recovery times of 78/31 s, respectively. More recently, a strategy to develop high-performance H₂S sensors operable at room temperature has been proposed by integrating two-dimensional MoS₂ with spinel-structured ZnCo₂O₄ [30]. The resulting MoS₂@ZnCo₂O₄ nanocomposite demonstrated a fast response/recovery times of 12/28 s to 10 ppm H₂S, along with a low detection limit of 0.5 ppm and long-term stability exceeding 28 days. These enhancements were attributed to the high specific surface area of MoS₂ and the effective charge transfer enabled by the p–n heterojunction between the two components.

Inorganic composite hydrogen gas sensors have also improved sensing performance by integrating noble metals or metal oxides in a synergistic manner [159–151]. A representative hydrogen gas sensor utilizes a composite structure of LaAlO₃ (LAO) and SrTiO₃ [11], which form a two-dimensional electron gas (2DEG) heterointerface, further modified with Pd nanoparticles [150]. In this structure, Pd catalyzes the dissociation of hydrogen molecules, and the resulting hydrogen atoms modulate the carrier density of the 2DEG at the LAO/STO interface, inducing a change in conductance. The Pd/LAO/STO sensor demonstrated a response/recovery times of 48/57 s, respectively, to 20 ppm H₂ at 80 °C. Another approach for hydrogen gas detection employs CeO₂-loaded In₂O₃ hollow spheres, as illustrated in Fig. 4c [139]. While In₂O₃ is an n-type semiconductor capable of gas sensing on its own, the incorporation of CeO₂ forms a heterojunction that facilitates electron transfer from CeO₂ to In₂O₃, resulting in an electron accumulation layer. This promotes increased oxygen adsorption and ionization, thereby amplifying the resistance change upon exposure to hydrogen gas. With 2 at% CeO₂ loading, the sensor exhibited rapid response/recovery times of 1/9 s, respectively. 

One representative approach for the effective detection of acetone gas involves the use of ZnO- and SnO_2_- based inorganic composites [131–136]. For instance, a p–n heterojunction formed by combining n-type ZnO hollow spheres with p-type NiO nanoparticles exhibits a significantly increased initial resistance in air due to electron depletion at the surface [138]. Upon exposure to acetone gas, the adsorbed oxygen ions react with the target gas, releasing electrons back into the conduction band, thereby increasing conductivity. This results in a rapid resistance change, and the sensor demonstrates a fast response/recovery times of 1/20 s under 100 ppm acetone at 275 °C. To address the challenge of high operating temperatures, a structure incorporating Au nanoparticles on ZnO nanorods has been proposed as shown in Fig. 4d [145]. Au, possessing a higher work function than ZnO, withdraws electrons at the junction, forming Schottky barriers and increasing electron trapping in air, which raises the initial resistance. Upon introduction of acetone, the adsorbed oxygen ions react with the gas, releasing electrons back to the ZnO, leading to a sharp decrease in resistance and a strong sensing signal. Owing to this structural enhancement, the Au@ZnO sensor achieved excellent sensing performance, exhibiting a rapid response/recovery times of 1/20 s under 100 ppm acetone even at a relatively low temperature of 172 °C. Meanwhile, a core–shell structured sensor composed of spinel ZnFe₂O₄ and ZnO has attracted attention as a promising material for acetone detection [140]. Although it operates at a relatively high temperature of 320 °C and exhibits a slower response time of 14 s, it demonstrates excellent sensitivity and selectivity. The formation of an n–n heterojunction between ZnO and ZnFe₂O₄ enables effective charge transport modulation, while the hollow architecture increases the reactive surface area, allowing for ppb-level detection of acetone. The sensor also exhibits stable response behavior over repeated sensing cycles.

Unlike reducing gases such as hydrogen and acetone, NO₂ exhibits a high electron affinity and strong oxidizing ability, allowing it to readily capture electrons from the surface of metal oxides [144, 143]. As a result, NO₂ sensing reactions can be activated at relatively low temperatures [149, 147]. A representative example is a nanoheterojunction-based sensor constructed by partially oxidizing SnS₂ nanoflowers to form SnO₂ [149]. The n–n heterojunction between SnS₂ and SnO₂ facilitates charge transfer, amplifying the resistance change upon NO₂ adsorption and thereby enhancing sensitivity. Additionally, the flower-like hierarchical architecture increases the specific surface area, promoting gas adsorption and diffusion. This sensor demonstrated a high response of 51.1 toward 1 ppm NO₂ at 100 °C, with a detection limit below 125 ppb. Another NO₂ gas sensor features a heterostructure of two-dimensional Ti₃C₂Tₓ and ZnO nanorods, as shown in Fig. 4e [152]. At their interface, multiple Schottky barriers are formed, enhancing electron trapping during NO₂ adsorption and resulting in a significant resistance change. Ti₃C₂Tₓ also acts as an efficient electron transport channel, improving response speed. The sensor showed a high response of 190% to 50 ppm NO₂ at 80 °C, with a response time under 13 s and excellent selectivity and stability. A room-temperature NO₂ sensor was developed using an Au-decorated In₂S₃/In₂O₃ microflower heterostructure [153]. The heterojunction between In₂S₃ and In₂O₃ facilitates charge transfer through electron redistribution, while Au nanoparticles enhance surface oxygen ionization and electron trapping, leading to improved sensitivity. The sensor exhibited fast response/recovery times of 12/27 seconds to 100 ppm NO₂ and maintained stable performance under 90% relative humidity with high selectivity. Overall, inorganic composite architectures demonstrate that interfacial engineering and heterojunction design can significantly enhance sensitivity and selectivity, suggesting a promising route toward room-temperature operation and real-world olfactory applications.

**Table 3:** Sensing performance of various composite inorganic materials-based gas sensors

Sensing Gas	Sensing Materials	Detection Method/Technology	Working Temperature (℃)	Concentration (ppm)	Response Time (s)	Recovery Time (s)	Reference
Ethylene	ZnO@Ag	Chemiresistive	Room Temp.	30	300	600	[130]
	CeO_x_-SnO_2_	Chemiresistive	350	0.3	5.18	10	[131]
	Cr_2_O_3_-SnO_2_	Chemiresistive	350	2.5	6	69	[132]
	Pd-SnO_2_	Chemiresistive	250	100	1	103	[133]
H_2_S	ZnO-Cr_2_O_3_	Chemiresistive	350	150	-	940	[134]
	GQD-SnO_2_/ZnO	Chemiresistive	Room Temp.	0.1	14	13	[135]
	In_2_O_3_/Ag	Chemiresistive	Room Temp.	0.1	84	186	[136]
	MoS_2_ − ZnCo_2_O_4_	Chemiresistive	Room Temp.	10	12	28	[30]
	ZnO-GaN	Chemiresistive	220	50	78	31	[137]
H_2_	Pd/SnO_2_	Chemiresistive	125	10	1	22	[138]
	CeO_2_/In_2_O_3_	Chemiresistive	160	50	1	9	[139]
	TiO_2_-α-Fe_2_O_3_	Chemiresistive	120	50	25	48	[140]
	LaAlO_3_/SrTiO_3_	Chemiresistive	80	20	48	57	[141]
	Cs_x_WO_3_/MoS_2_	Chemiresistive	Room Temp.	500	60	120	[142]
Acetone	Co_3_O_4_/SnO_2_	Chemiresistive	350	100	0.62	46.5	[143]
	In/WO_3_-SnO_2_	Chemiresistive	200	1	4	2	[144]
	Au-ZnO	Chemiresistive	172	100	1	20	[145]
	NiO-ZnO	Chemiresistive	275	100	1	20	[146]
	SnO_2_-ZnO	Chemiresistive	240	0.82 ppb	108	44	[147]
	ZnO-ZnFe_2_O_4_	Chemiresistive	250	100	56	89	[148]
NO_2_	Fe@In_2_O_3_	Chemiresistive	100	1	276	150	[149]
	Au@In_2_S_3_/In_2_O_3_	Chemiresistive	Room Temp.	100	12	27	[150]
	SnS_2_-SnO_2_	Chemiresistive	100	1	299	143	[151]
	Ti_3_C_2_T_x_ @ZnO	Chemiresistive	80	50	13	-	[152]
	NiO/CdS-CdO	Chemiresistive	150	100 ppb	47	66	[153]

## M13 bacteriophage carbon nanotubes hybrid materials-based gas sensors

Gas sensors based on organic and inorganic materials each offer distinct advantages; however, they also exhibit inherent limitations. Organic material-based sensors are characterized by excellent selectivity and the potential for diverse surface functionalization. Therefore, hybrid platforms that integrate organic and inorganic elements represent a promising pathway to overcome these trade-offs and achieve balanced, high-performance gas sensing. Nonetheless, they often suffer from limited durability, as well as poor thermal and chemical stability. In contrast, inorganic material-based sensors typically demonstrate superior durability and electrical properties [161–165]; however, their selectivity is generally low, and achieving complex surface functionalization remains challenging.

To address these limitations, hybrid gas sensors that integrate both organic and inorganic components have emerged as a promising solution. Such hybrid systems effectively combine the high selectivity of organic materials with the excellent sensitivity and durability of inorganic materials, thereby overcoming the performance constraints of conventional single-component sensors. Furthermore, hybrid structures enable enhanced sensitivity and selectivity, along with rapid response/recovery characteristics toward a wide range of target gases.

Hybrid material systems are increasingly adopted to resolve the intrinsic trade-offs of single-component gas sensors, which often struggle to simultaneously achieve high selectivity, high sensitivity, and low-power operation. A representative example is the integration of M13 bacteriophage (M13) with carbon nanotubes (CNT). The M13 serves as a genetically programmable recognition element, capable of binding target gas molecules with exceptional precision, but its low electrical conductivity limits direct signal transduction. CNT, in contrast, provide a highly conductive, large-surface-area network that amplifies electrical changes, yet they lack intrinsic selectivity. When combined, the M13 layer concentrates and orients target analytes directly onto the CNT network, enabling rapid and efficient charge transfer at the bio–nano interface. This cooperative mechanism not only amplifies the sensing signal but also ensures that it is dominated by specific molecular interactions rather than nonspecific adsorption. Critically, such hybrid architectures deliver robust, selective responses at room temperature—overcoming the high-power thermal activation required by many metal oxide sensors—and illustrate the broader principle that coupling a selective recognition layer with an efficient transducer pathway is a powerful strategy for next-generation gas sensing.

In this section, particular attention is given to a hybrid sensing platform composed of the organic M13 and the inorganic CNT. The subsequent section discusses the individual properties of the M13 and CNT, and explores their synergistic integration within hybrid gas sensing systems.

### M13 bacteriophage (M13) based gas sensors

Recent studies have demonstrated the successful application of M13-based electronic nose (e-nose) platforms in various practical fields, including breath gas analysis and food quality monitoring [166–169]. This broad applicability arises from the unique morphological, biochemical, and functional properties of the M13. Unlike conventional inorganic or organic materials, the M13 is a bio-derived nanomaterial that possesses both biomolecular recognition capability and self-assembly characteristics [170–172]. Structurally, the M13 possesses a linear filamentous architecture approximately 880 nm in length and 8.8 nm in diameter, resulting in an exceptionally high surface-to-volume ratio. These structural features enable the dense arrangement of diverse functional groups on the sensor surface, as each phage displays about 2,700 copies of the pVIII protein, which are ideally suited for surface modification and conjugation with functional molecules [173–177]. Such structural characteristics facilitate precise molecular interactions with a wide range of volatile organic compounds (VOCs), playing a pivotal role in enhancing sensor sensitivity. Indeed, M13-based sensors have achieved detection limits for VOCs in the ppb range, thereby demonstrating their potential as high-sensitivity electronic nose technologies [166, 167]. Furthermore, the phage display technique enables the site-specific presentation of selective peptide or receptor sequences on the phage surface. By screening large peptide libraries—comprising billions of candidates—high-affinity and target-specific binding motifs can be identified and incorporated, allowing the development of highly selective and customized sensor platforms [178–180]. The M13 serves as a genetically programmable biological recognition element, offering a level of molecular specificity that surpasses many synthetic organic and inorganic receptors. Its surface can be engineered to display tailored peptide sequences, enabling targeted binding to a wide range of analytes—a capability that directly addresses the limited selectivity of many single-material gas sensors. This precise molecular recognition stems from the phage’s ability to self-assemble into ordered nanostructures with controlled binding site density, which in turn modulates interfacial charge distribution and optical properties when integrated into a transducer platform. Recent advances have extended the impact of M13 beyond gas sensing: genetically engineered phages have been employed as ultrasensitive platforms for capturing rare circulating tumor cells to enable precise cancer subtyping [181], and as targeted immunotherapeutic agents for cancer treatment [182].When incorporated into hybrid architectures with conductive nanomaterials, such as CNT, M13 provides unparalleled selectivity while maintaining operational stability under ambient conditions. In addition, the M13 exhibits excellent chemical stability and mechanical robustness, maintaining reliable sensing performance under diverse environmental conditions. Simultaneously, its inherent self-assembly properties allow for the formation of well-ordered nanostructures, contributing to the structural uniformity of the sensor surface and ensuring signal reproducibility [174]. Finally, the M13 can be readily hybridized with various functional nanomaterials, such as conductive polymers and metal nanoparticles, enabling the fabrication of composite sensors. These hybrid systems can provide both electrochemical and optical signal amplification, offering promising prospects for liquid sample analysis and the development of highly sensitive, real-time detection platforms.

**Fig. 5:**
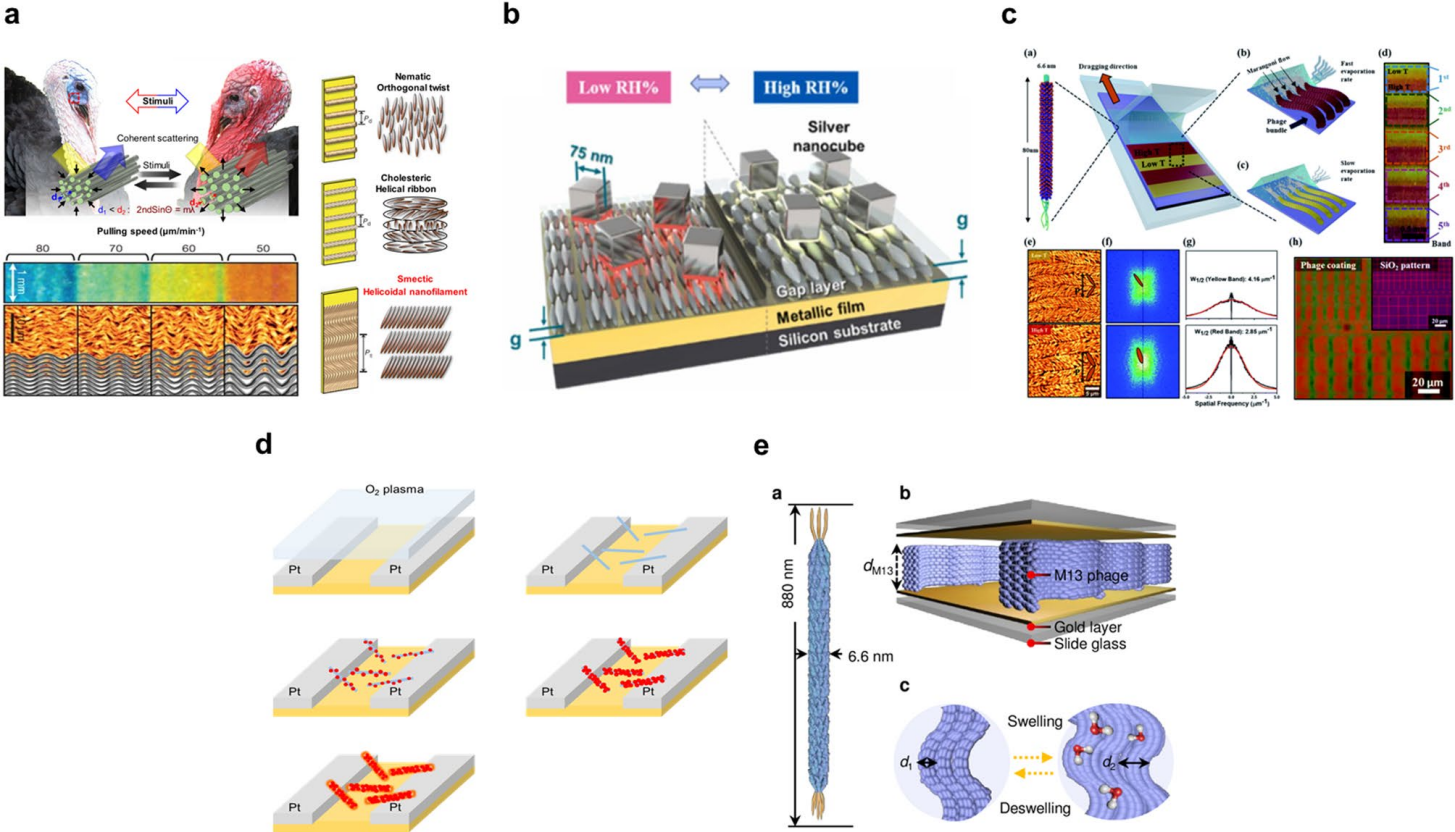
Representative gas sensing mechanisms of M13 bacteriophage-based hybrid sensors for various analytes. **a** M13 bacteriophage-based VOC sensing via structural color changes in self-assembled nanostructured thin films with ppb-level sensitivity. Reproduced with permission from [183]. Copyright 2011, Springer Nature. **b** Plasmonic VOC sensing using genetically engineered M13 bacteriophage layers between silver nanocubes and mirrors, modulating plasmonic gaps and visible color shifts upon VOC binding. Reproduced with permission from [184]. Copyright 2022, Elsevier. **c** Colorimetric VOC detection through M13 bacteriophage aligned on micropatterned substrates, enabling tunable bundle diameter and rapid target-specific color changes via genetic modification. Reproduced with permission from [185]. Copyright 2021, Royal Society of Chemistry. **d** Nanowire-peapod NH₃ sensor templated by gold-specific M13 bacteriophage exhibiting irreversible resistance increase upon ammonia exposure with a 0.005 ppm detection limit. Adapted with permission from [186]. Copyright 2016, IOP Publishing. **e** Fabry–Pérot etalon gas sensor using M13 bacteriophage as tunable spacers that swell in response to humidity and VOCs, altering cavity distances and shifting reflected light wavelengths for sensitive detection. Reproduced from [187], licensed under CC BY. Copyright 2024, Optica Publishing Group

As shown in Fig. 5a, illustrates a self-assembled M13-based optical sensor was developed to mimic the structural color change observed in turkey skin. By adjusting the drawing speed, nanostructured thin films were fabricated that exhibit color shifts upon VOC exposure. The sensor demonstrated ppb-level sensitivity and successfully distinguished between healthy individuals and lung cancer patients through breath analysis [183]. A plasmonic VOC sensor was created using genetically engineered M13 positioned between silver nanocubes and a mirror film as illustrated in Fig. 5b. The M13 layer swells or contracts upon VOC binding, modulating the plasmonic gap and shifting the visible color. This sensor achieved ppb-level sensitivity and over 89% accuracy in lung cancer patient classification [184]. A colorimetric sensor was developed by aligning M13 on micro-patterned substrates using meniscus-dragging deposition as shown in Fig. 5c. The structural color varied with bundle diameter, which was tunable via temperature and drawing speed. The sensor rapidly responded to humidity and chemicals, showing visible color shifts within 1 s and target-specific responses enabled through genetic modification [185]. As illustrated in Fig. 5d, a nanowire-peapod NH₃ gas sensor was fabricated using gold-specific M13 to template AuNPs coated with polypyrrole. NH₃ exposure caused irreversible increases in resistance due to disruption of the polymer backbone. The sensor achieved a low detection limit of 0.005 ppm, highlighting the phage’s templating advantage [186]. A Fabry–Pérot etalon was constructed using M13 as tunable spacers as shown in Fig. 5e. The M13 layer swelled in response to humidity and VOCs (ethanol, IPA), altering cavity distances and shifting the wavelength of reflected light. With detection limits down to the ppb range and high measurement precision (± 0.03 nm), the system enables sensitive, real-time gas sensing [187]. Together, these case studies highlight the versatility of M13 as a bio-derived platform, capable of enabling both optical and electrical transduction pathways for real-time gas sensing Representative sensing performances of M13-based gas sensors are summarized in Table 4.

Although M13-based gas-phase sensors exhibit significant potential and offer high sensitivity and selectivity due to the unique biological properties of the M13, several critical limitations remain for their practical application. M13-based devices—spanning gas-phase sensors and optoelectronic platforms—offer high sensitivity and selectivity thanks to genetically programmable assembly and nanoscale structural control [188, 189]. Nevertheless, several practical limitations must be addressed for real-world deployment. First, environmental robustness and reproducibility are concerns: as a biologically derived material, M13 is sensitive to temperature, humidity, and pH, which can undermine long-term stability and batch-to-batch consistency. Because gas sensors must operate across diverse conditions, strategies to harden the material are essential. Second, M13’s intrinsically low electrical conductivity hampers efficient signal transduction when responses are evaluated via conductivity changes upon gas adsorption. Third, in complex mixtures, interference can erode selectivity; despite advances in genetic engineering and machine-learning-assisted analysis, real-time, high-precision deconvolution remains challenging. To address these issues, recent work has focused on hybrid materials that combine M13 with functional nanomaterials (metal nanoparticles, carbon nanomaterials, metal oxides, polymers, and other biomaterials). Among them, integrating M13 with conductive carbon nanotube (CNT) networks is particularly promising: ordered nanostructures with tailored molecular recognition from M13, coupled with percolating charge-transport pathways from CNTs, enable precise tuning of optical and electrical properties and improve overall sensing robustness and performance.

**Table 4:** Sensing performance of various M13 bacteriophage-based gas sensors

Sensing Gas	Sensing Materials	Detection Method/Technology	Working Temperature (℃)	Concentration (ppm)	Response Time (s)	Recovery Time (s)	Reference
VOCs	M13	Optical	Room Temp.	0.3	< 20	-	[178]
VOCs, Humidity	M13/AuNPs	Optical	Room Temp.	Humidity < 20%	1	1	[179]
Humidity	M13	Optical	Room Temp.	Humidity < 20%	-	-	[180]
NH₃	M13/Au/Polypyrrole	Electrical	Room Temp.	0.005	< 900	-	[181]
Ethanol, IPA, Humidity	M13	Optical	Room Temp.	0.158	-	-	[182]

### Carbon nanotubes (CNT) hybrid materials-based gas sensors

CNT have garnered significant attention as next-generation materials [190–192] for highly sensitive gas sensors, owing to their unique electronic structure, exceptional mechanical strength, thermal stability, and large specific surface area. Their one-dimensional electron transport characteristics and high surface reactivity make them extremely responsive to gas molecule interactions, thereby enabling real-time detection at low concentrations.

Recent advances in CNT-based gas sensors have achieved outstanding performance, including sub-ppb detection limits and millisecond-level response times in flexible and miniaturized device architectures [193, 194]. These achievements highlight the high electrical conductivity, large surface-to-volume ratio, and reliable scalability of CNT platforms, making them promising candidates for high-performance gas sensing in real-time and wearable olfactory display applications.

**Fig. 6:**
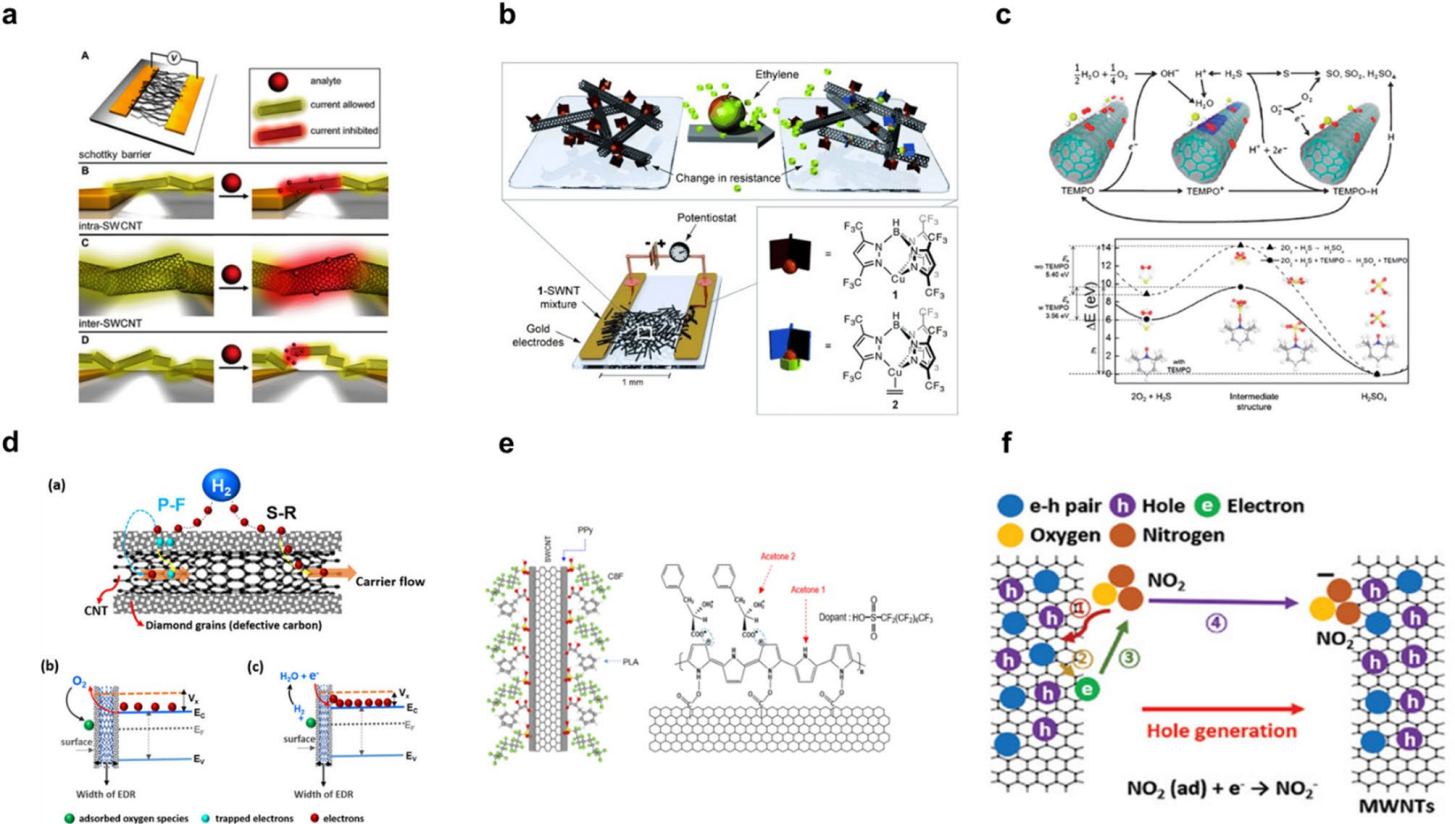
Representative gas sensing mechanisms of CNT-based hybrid sensors for various analytes. **a** General sensing mechanisms in CNT-based gas sensors, illustrating three pathways: (1) charge carrier modulation within CNTs (intra-CNT), (2) tunneling conduction at inter-CNT junctions (inter-CNT), and (3) Schottky barrier tuning at the metal–CNT interface. Reproduced with permission from [195]. Copyright 2015, John Wiley and Sons. **b** Ethylene sensing using Cu(I)-complex functionalized SWCNT: Ethylene binding suppresses doping from the Cu(I) complex, decreasing charge carrier density in the CNT. Reproduced with permission from [196]. Copyright 2012, John Wiley and Sons. **c** H₂S detection with TEMPO-functionalized CNT: Redox reactions between TEMPO and H₂S generate electrons that dope the CNT network, reducing hole concentration and decreasing conductivity. Reproduced with permission from [197]. Copyright 2013, Royal Society of Chemistry. **d** Hydrogen sensing using nanodiamond-induced CNT (NDCNT): The sp³-rich outer shell facilitates hydrogen adsorption and desorption, enabling a Poole–Frenkel and Schottky–Richardson transport mechanism with fast response and long-term stability. Reproduced with permission from [198]. Copyright 2021, American Chemical Society. **e** Acetone detection using PLA/C8F-doped PPy/SWCNT composites: Acid–base interactions between PLA and acetone promote hole accumulation in the PPy layer, enhancing intra- and inter-CNT conductivity. Reproduced with permission from [199]. Copyright 2022, MDPI. **f** NO₂ sensing with flexible MWCNT sensors: NO₂ acts as an electron acceptor, increasing hole concentration in p-type CNT and leading to enhanced conductivity. Reproduced with permission from [200]. Copyright 2019, John Wiley and Sons

The sensing mechanisms of CNT-based gas sensors can be broadly classified into three categories: (1) variation in charge density within the CNT (intra-CNT effect); (2) modulation of tunneling conductivity at the junctions between CNT (inter-CNT effect); and (3) tuning of the Schottky barrier at the metal–CNT interface (Schottky barrier modulation). These mechanisms operate independently or synergistically depending on the device structure and the target gas, and a conceptual illustration of these processes is shown in Fig. 6a [195]. CNT function as the high-performance transducer element in M13–CNT hybrid sensors, effectively addressing the non-conductive nature of the phage. They provide ultrahigh electrical conductivity, a large surface-to-volume ratio, mechanical flexibility, and reliable scalability for device fabrication. Unlike purely organic sensors, CNT maintain high signal fidelity and robustness over long-term operation, and unlike many inorganic sensors, they can achieve comparable or superior sensitivity without high-temperature activation. In hybrid form, CNT amplify the selective molecular interactions of the M13 layer by facilitating rapid, efficient charge transfer at the bio–nano interface, minimizing recombination losses and enhancing signal-to-noise ratios. This synergy—combining the M13’s targeted molecular capture with the CNT network’s efficient signal transduction—yields sensors capable of robust, room-temperature operation with rapid response, high sensitivity, and exceptional selectivity. Such performance represents a significant advancement over conventional single-material platforms and underscores the promise of hybrid materials for next-generation, real-time, wearable olfactory display technologies.

As shown in Fig. 6b, for ethylene sensing, functionalization with metal complexes enables interaction with this nonpolar gas. A Cu(I) complex with a fluorinated tris(pyrazolyl)borate ligand was incorporated onto SWCNT to control doping states. Upon ethylene binding, the interaction weakens, suppressing doping and reducing conductivity via an intra-CNT doping inhibition mechanism. The sensor exhibited linear response characteristics in the 0.5–50 ppm range and sensitivity below 1 ppm. Enhanced reactivity was also observed for (6,5) chirality SWCNT, and the system proved useful for monitoring ethylene evolution during fruit ripening [196]. In contrast, a Pd–V₂O₅–TiO₂-based cascade sensor uses ethylene oxidation to acetaldehyde via a Wacker-type mechanism, followed by its reaction with hydroxylamine hydrochloride to generate HCl gas. This HCl acts as a strong p-type dopant for SWCNT, inducing an increase in conductivity. The system achieved detection down to 0.2 ppm within 5 min, with high selectivity enabled by a dual-sensor configuration [201].

Hydrogen sulfide (H₂S) decreases the hole concentration in CNT by acting as an electron donor, thereby lowering conductivity. A TEMPO-functionalized SWCNT sensor utilizes redox reactions between TEMPO⁺ and H₂S to generate electrons that dope the CNT, reducing p-doping and decreasing the current. Sulfur oxidation byproducts re-adsorb onto the surface, further modulating the electrical signal. This structure achieved a 420% increase in response under 60% RH and reduced the activation energy by 34% due to TEMPO’s catalytic effect as shown in Fig. 6c [197]. Additionally, a CNT film coated with amorphous Fe₂O₃ demonstrated ultrahigh sensitivity down to 2 ppb at room temperature. The broad surface area and disordered oxide morphology enhanced H₂S adsorption and charge transfer. The device exhibited response/recovery times of 127/563 s at 100 ppb, respectively, and its flexible, fiber-convertible structure supports integration into wearable sensors [202]. H₂S-selective CNT sensors have also been demonstrated [203], and Au–Pd decorated CNT hybrids enabled simultaneous detection of H₂S and Nitrogen dioxide (NO₂) [204].

For H₂ sensing, a representative structure is based on CNT grown using a nanodiamond-induced method. The sp³-carbon-rich outer shell facilitates H₂ adsorption and desorption, while the H₂ spillover mechanism, combined with Poole–Frenkel and Schottky–Richardson conduction models, contributes to fast response and long-term stability as shown in Fig. 6d [198]. Additionally, Pd-doped SWCNT sensors utilize the formation of PdH_x_ to modulate the electronic structure at the metal–CNT interface, thus enabling Schottky barrier modulation. These devices demonstrated rapid response and stable room-temperature operation over a hydrogen concentration range of 20–20,000 ppm [205]. Moreover, Pd/CNT composites showed n-type transition and Seebeck potential effects, indicating a hybrid mechanism involving both intra-CNT and interfacial contributions [206].

In the case of acetone detection, both polymer-based and metal oxide-based CNT composites operate primarily via the intra-CNT mechanism, involving modulation of the hole concentration within the CNT. A PLA/C8F-doped polypyrrole (PPy)/SWCNT composite enables selective adsorption through acid–base interactions between the –COO⁻ groups of PLA and the proton-donating hydrogens of acetone, leading to increased hole concentration and enhanced conductivity. This structure enables stable detection at 50 ppb under various humidity conditions as shown in Fig. 6e [199]. In contrast, metal oxide-based sensors exploit electron transfer and heterojunction formation at the CNT–oxide interface. A flower-like α-Fe₂O₃/MWCNT structure facilitates the oxidation of acetone at the oxide surface, generating electrons that are injected into the CNT. This process results in intra-CNT conductivity modulation and yields a response time of 2.3 s and a response value greater than 20 at 50 ppm. Furthermore, Fe₂O₃ nanoparticle-doped CNT composites exhibited a response value of 2.34 at 80 ppm, with a rapid response time of 4–6.5 s and outstanding stability under 0.6 wt% doping at 220 °C [207].

Beyond these examples, many other studies have also demonstrated CNT-based composites for acetone detection [208–211].

NO₂, a strong electron acceptor, extracts electrons from the CNT, thereby increasing the hole concentration and enhancing conductivity—an effect governed by the intra-CNT mechanism. A flexible MWCNT-based sensor exemplifies this behavior, offering room-temperature operation, reproducibility, and a fast response in the 10–50 ppm range (Fig. 6f) [200]. Furthermore, SnO₂ nanoparticle-doped CNT composites form p—n heterojunctions, where NO₂ adsorption at the oxide interface promotes hole accumulation in the CNT, enabling detection down to 20 ppb [212]. Similarly, S-doped SWCNT showed a 54.2% improvement in sensitivity due to enhanced charge transfer, while maintaining high stability and resistance to humidity effects [213]. In addition to these, numerous other studies have reported on NO₂ detection using CNT-based sensors, further demonstrating the adaptability of CNTs in gas sensing applications Representative sensing performances of CNT-based gas sensors are summarized in Table 5.

In summary, CNT hybrid-based gas sensors leverage diverse mechanisms—including charge transfer, doping modulation, and interfacial interactions—to achieve selective and sensitive detection tailored to the physicochemical properties of each target gas. These sensors continue to evolve toward multifunctional, wearable, and low-power applications in next-generation platforms.

**Table 5:** Sensing performance of various CNT-based gas sensors

Sensing Gas	Sensing Materials	Detection Method/Technology	Working Temperature (℃)	Concentration (ppm)	Response Time (s)	Recovery Time (s)	Reference
Ethylene	SWCNT + Calix[4]pyrrole	Chemiresistive	Room Temp.	20–500	-	-	[214]
	Pd–V₂O₅–TiO₂ + SWCNT	Chemiresistive	40	0.2–100	300 (100 ppm)	700 (100 ppm)	[201]
H_2_S	CNT (PAS) sensor	Chemiresistive	Room Temp.	33 ppb	fast	fast	[203]
	CNT/Fe₂O₃ Film	Chemiresistive	Room Temp.	2 ppb − 10 ppm	127 (100 ppb)	563 (100 ppb)	[202]
H_2_S, NO_2_	MWCNT_Au_Pd	Chemiresistive	45–200	ppb - ppm	fast	fast	[204]
H_2_	Pd/CNT composite	Chemiresistive	Room Temp.	2,500–25,000	-	-	[206]
	SWCNT + Pd NPs	Chemiresistive	25	20–20,000	10	3	[205]
Acetone	MWCNT/Co₃O₄	Chemiresistive	Room Temp.	10–1000	5	10	[208]
	MWCNT + Ag NPs	Chemiresistive	27	50–800	-	-	[209]
	MWCNT + ZIF-8	Chemiresistive	Room Temp.	0.62-40	30	30	[210]
	α-Fe₂O₃/MWCNT	Chemiresistive	220	5-800	2.3	-	[211]
NO_2_	SnO_2_/SWCNT	Chemiresistive	Room Temp.	10	50	60	[215]
	CNT + SnO_2_	Chemiresistive	Room Temp.	1	300	1,000	[212]
	MWCNT + SnO_2_	Chemiresistive	150	50 ppb	240	-	[216]
	CNT + ZnO	Chemiresistive	Room Temp.	10–50	3,500	1 ms	[217]
	SWCNT, S doping	Chemiresistive	Room Temp.	1 ppb-10 ppm	fast	fast	[213]
	CNTs, SnO₂, CuO	Chemiresistive	Room Temp.	10-ppb-1 ppm	4 min	10 min	[218]

### Next-generation applications and future prospects of AI-driven M13–CNT hybrid sensors

In the field of gas sensors, various emerging materials such as MOF, 2D nanomaterials (e.g., graphene, transition metal dichalcogenides), and block copolymers have recently attracted significant attention. MOF offer extremely high surface areas and tunable porosity, enabling excellent gas adsorption and selectivity, but their low electrical conductivity and insufficient structural stability hinder practical implementation. 2D nanomaterials possess outstanding electronic properties that enable sensitive and rapid gas detection, yet they are prone to restacking and aggregation, and face challenges in achieving uniformity and scalable production. Block copolymers are advantageous for nanoscale patterning and controlling selective binding sites, but their reliability can deteriorate due to vulnerability to long-term environmental fluctuations.

In comparison, constructing an M13–CNT hybrid material expected to allow the molecular-level design of specific binding sites tailored to target gases through genetic modification of the M13, thereby achieving very high selectivity. When combined with CNT networks, the hybrid anticipated to offer both excellent electrical conductivity and mechanical flexibility, and its self-assembly properties facilitate the fabrication of multi-channel sensor arrays. Furthermore, M13–CNT hybrids are projected to deliver stable and reproducible sensing performance under various environmental conditions and can be directly applied to real-time detection in complex devices or wearable olfactory displays.

Therefore, the M13–CNT hybrid platform is distinguished from existing MOF, 2D nanomaterials, and block copolymers by combining bioinspired selectivity, rapid and stable signal transduction, mechanical flexibility, and high integration potential, making it a next-generation, high-performance gas sensor material.

In parallel, recent advances in artificial intelligence (AI)-based data analysis and multi-gas discrimination technologies have emerged as key innovations in the fields of olfactory displays and gas sensors. Machine learning and deep learning algorithms play an important role in effectively interpreting high-dimensional sensor signals, enabling precise identification of various gases, compensating for sensor drift, and performing real-time classification of complex odor patterns.

Major techniques include traditional ML methods such as principal component analysis (PCA), linear discriminant analysis (LDA), k-nearest neighbors (kNN), and support vector machines (SVM), as well as the use of convolutional neural networks (CNN) for pattern recognition and long short-term memory (LSTM) networks for time-series data classification. For example, CNN–LSTM hybrid models can simultaneously analyze spatial and temporal information obtained from electronic nose (e-nose) sensor arrays, contributing to improved accuracy in multi-gas discrimination in complex environments. In addition, neural network–based models have demonstrated high performance in quantitative prediction (concentration estimation) and early warning of malodor or hazardous gases [219].

Recently, intelligent olfactory systems combining sensor hardware with AI signal processing have been introduced, with ultra-compact sensor arrays integrated with real-time AI inference extending applications to human–machine interaction, medical diagnostics, environmental monitoring, and immersive VR/AR environments. For instance, wearable olfactory interfaces employing deep learning-based gas recognition technologies have demonstrated significantly improved performance, including sub- to few-hundred-millisecond response times, simultaneous multi-odor output, and high energy efficiency [220].

From a forward-looking perspective, the combination of sensor array development and AI signal processing is expected to become increasingly sophisticated, resulting in real-time olfactory display platforms capable of maintaining high selectivity and stability even in complex gas mixtures. In particular, the adoption of advanced AI techniques such as transfer learning, domain adaptation, and multi-modal sensor fusion is anticipated to substantially improve data scarcity problems and adaptability to changing environmental conditions [221].

In summary, data-driven AI signal analysis and multi-gas discrimination technologies constitute an essential foundation for realizing next-generation olfactory displays, and the co-evolution of sensor hardware and AI algorithms will play a pivotal role in advancing the intelligence and practical implementation of future digital olfactory interfaces.

## Conclusions

This review has highlighted the significant progress in gas sensing materials for the advancement of olfactory display systems. Organic materials provide flexibility, biocompatibility, and tunable selectivity, whereas inorganic semiconductors deliver high sensitivity and long-term stability under harsh conditions. In particular, hybrid systems—exemplified by M13 bacteriophage–carbon nanotube composites—bridge the strengths of both approaches, combining bioinspired molecular recognition with efficient electrical transduction at room temperature. Together, these innovations illustrate how diverse sensing strategies are converging toward the stringent requirements of real-time, closed-loop olfactory display platforms.

Despite these advances, several hurdles remain for achieving multi-component odor representation. A primary challenge is cross-sensitivity in complex odor environments, where multiple volatile compounds generate overlapping responses. Overcoming this will require complementary sensor arrays combined with advanced data processing, including sensor fusion, adaptive filtering, and machine learning algorithms. Another key challenge is miniaturization and low-power integration for wearable applications, demanding compact multi-sensor modules, advanced packaging strategies, and wireless connectivity. Finally, long-term operational stability—particularly for organic and hybrid sensors—necessitates robust encapsulation, self-healing functional layers, and automated calibration protocols to mitigate drift and degradation.

Moving forward, three research directions appear essential: (i) the design of complementary sensor arrays with AI-driven signal deconvolution to mitigate cross-sensitivity; (ii) the development of compact, low-power integrated modules for wearable olfactory platforms; and (iii) the improvement of environmental stability through advanced encapsulation and self-healing materials. Pursuing these strategies will accelerate the realization of robust, real-time olfactory display systems and pave the way for their transition from conceptual prototypes to practical technologies with transformative applications in healthcare, environmental monitoring, and immersive human–machine interaction.

## Supplementary Information

Below is the link to the electronic supplementary material.

Supplementary Material 1

## Data Availability

Not applicable.
