# Supplementary material for: Recent progress of gas sensors toward olfactory display development
Source: Nano Converg. 2025 Aug 29;12:42. doi: 10.1186/s40580-025-00508-y (PMC12397036; doi:10.1186/s40580-025-00508-y)
Supplement: Supplementary file 1 — Supplementary Material 1 [file 40580_2025_508_MOESM1_ESM.docx]

**Permission Lists
Fig. 2a**


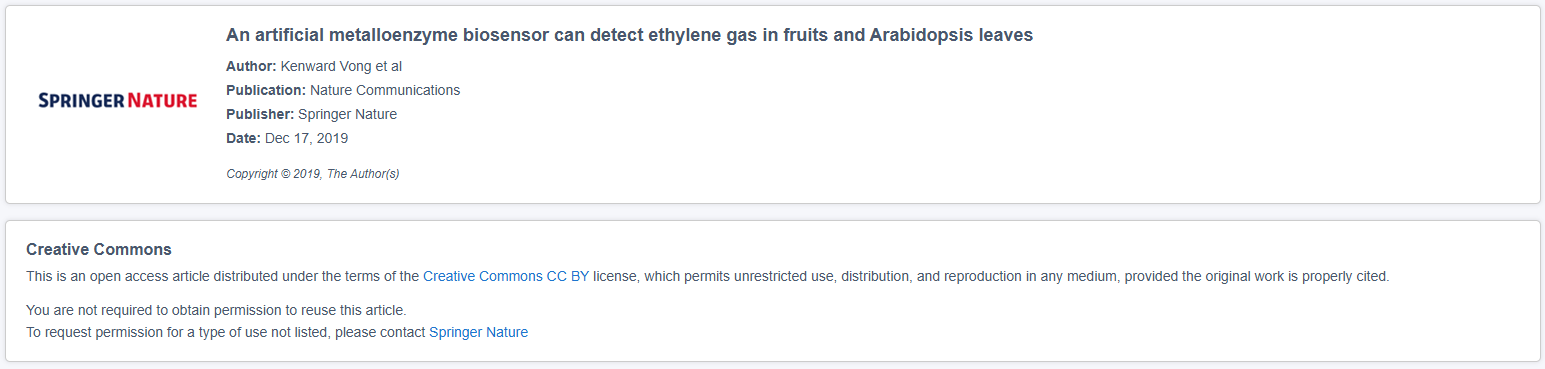
 **Fig. 2b**


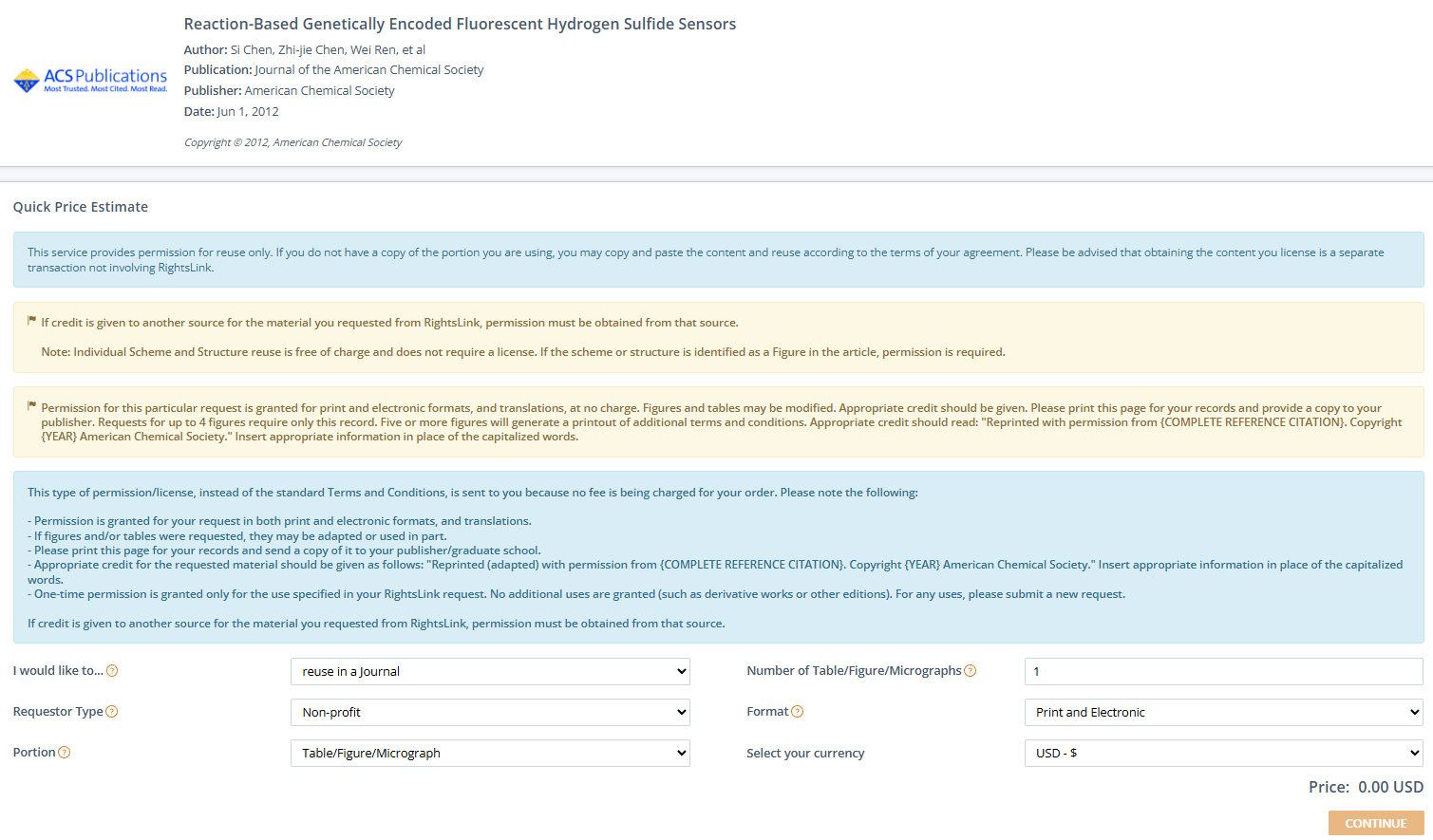


**Fig. 2c**


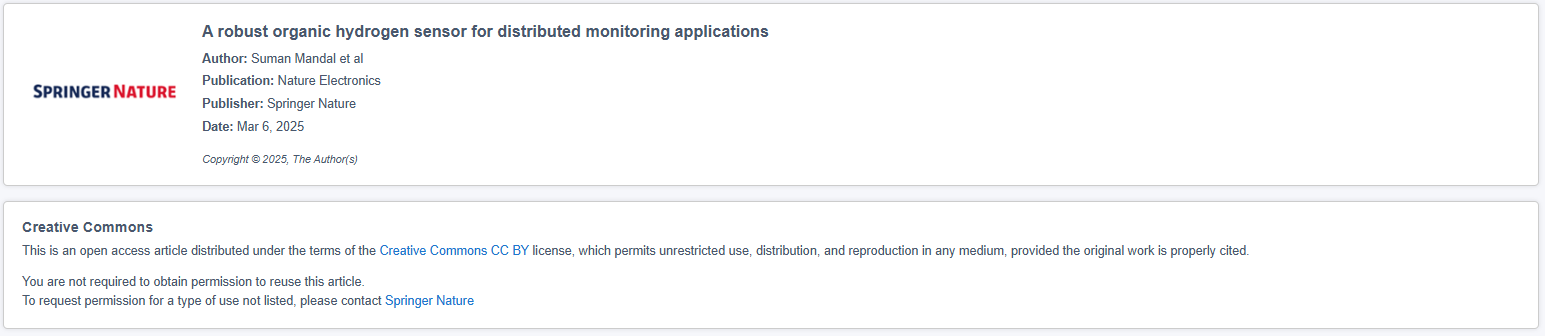


**Fig. 2d**


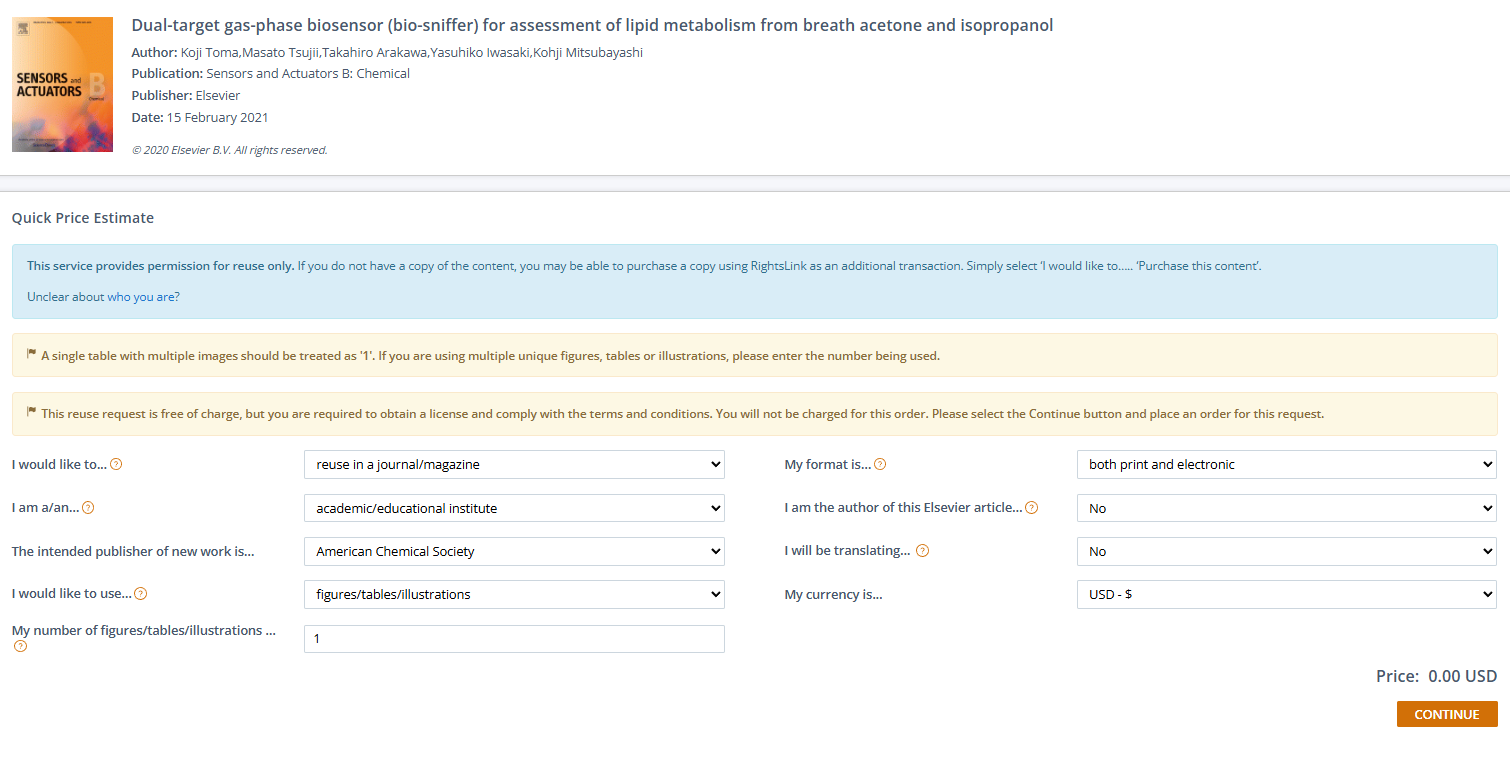


**Fig. 2e**


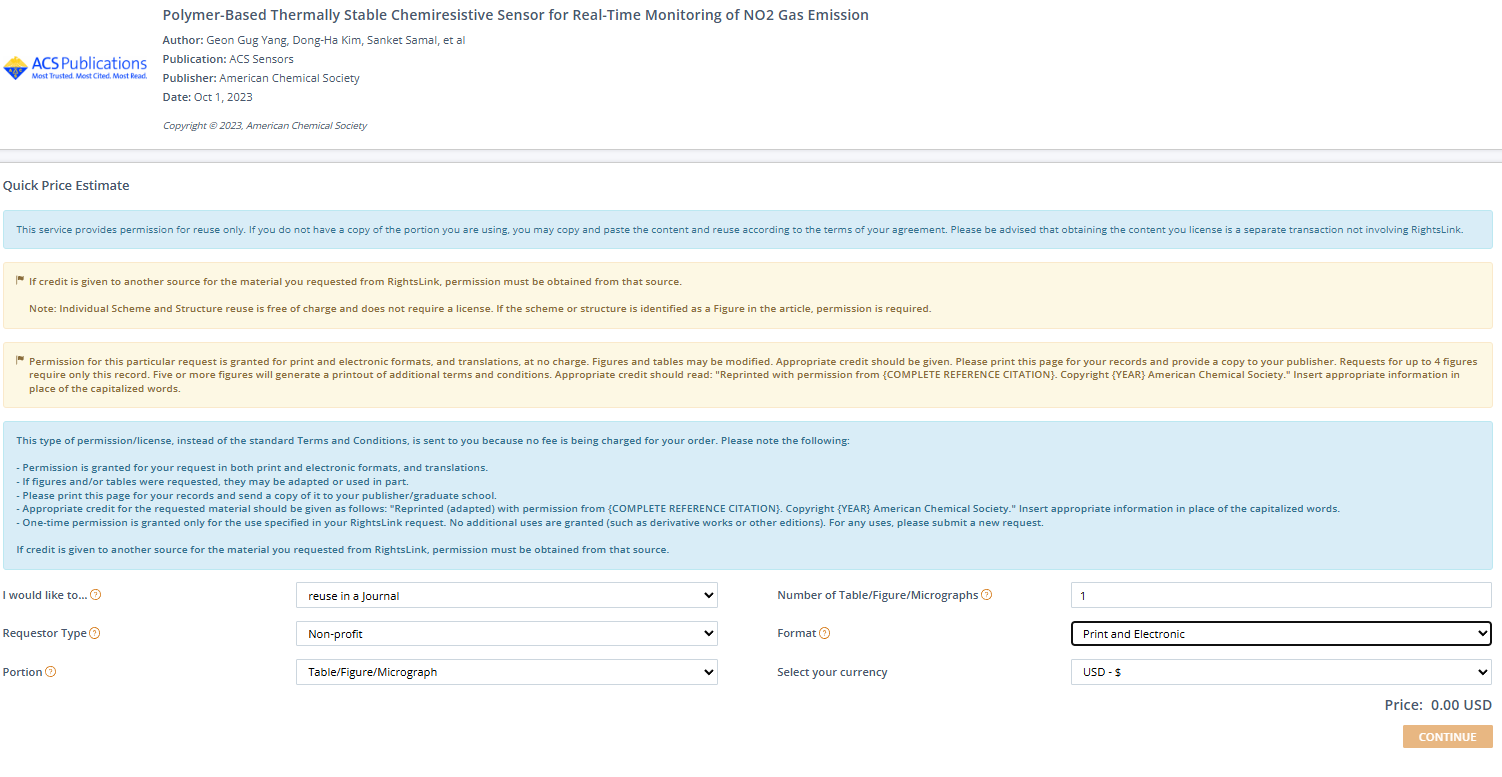


**Fig. 3a**


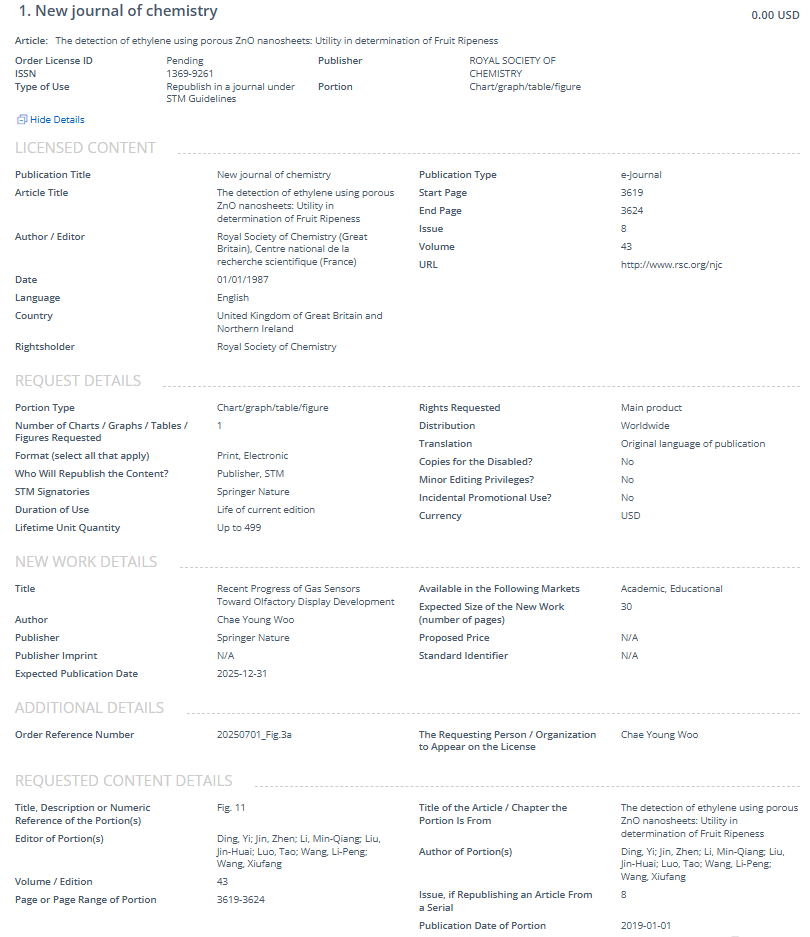


**Fig. 3b**


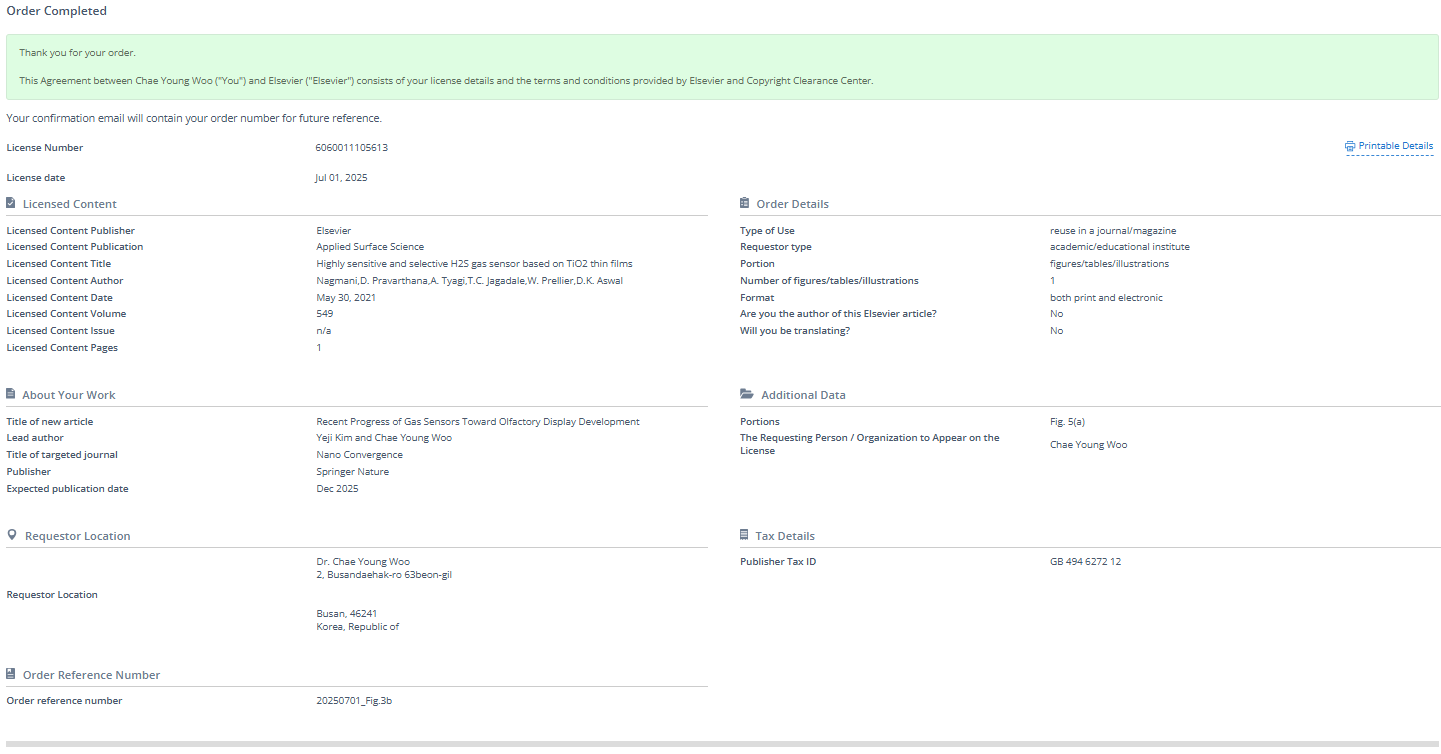


**Fig. 3c**


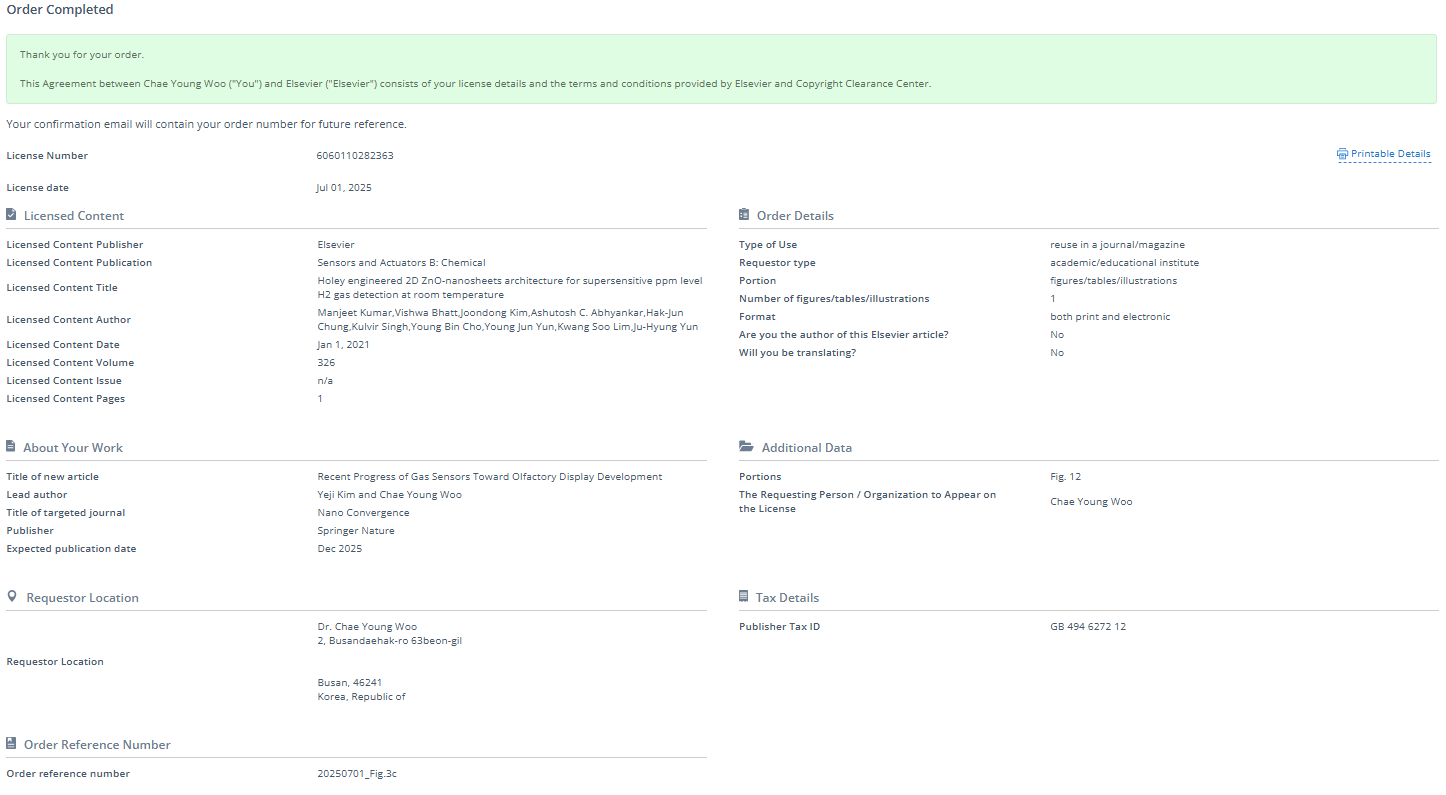


**Fig. 3d**


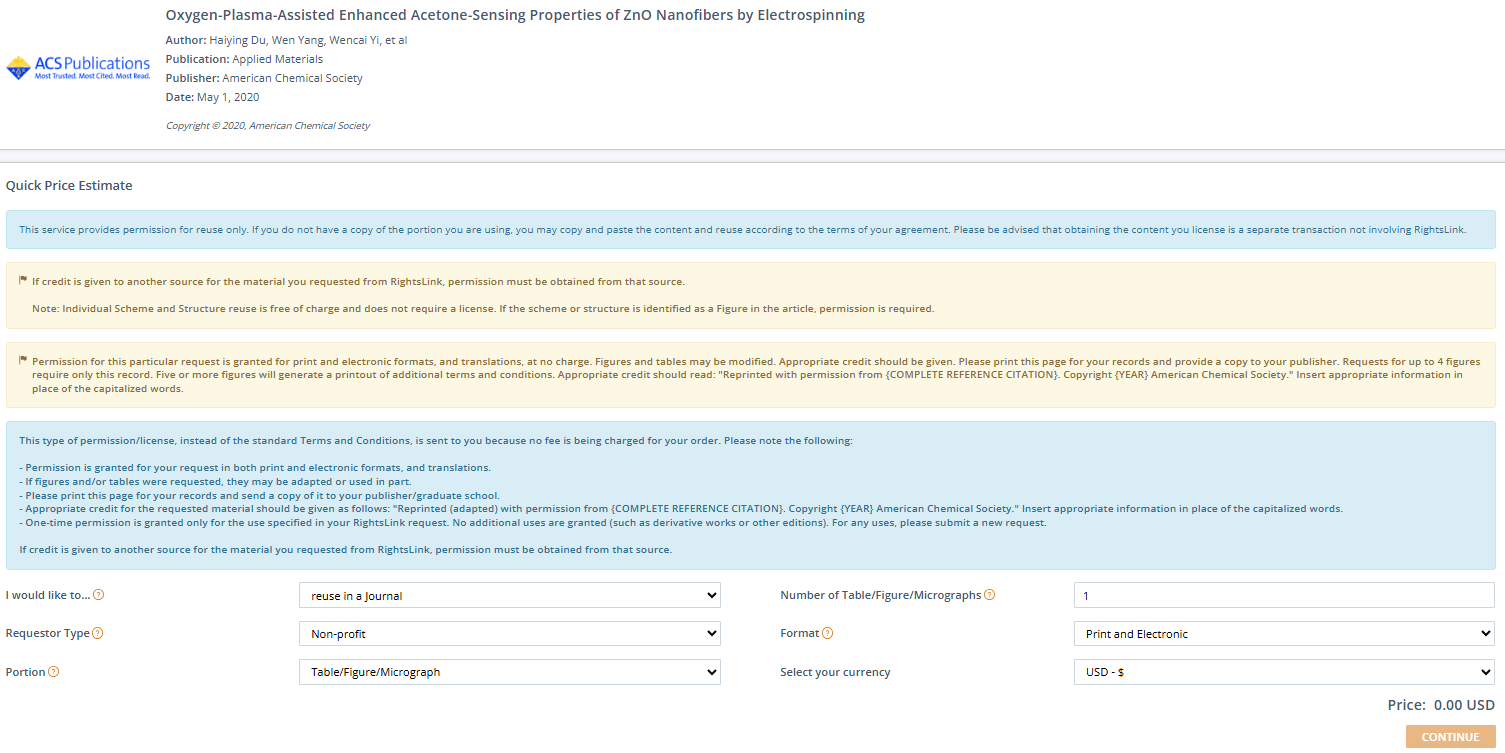


**Fig. 3e**


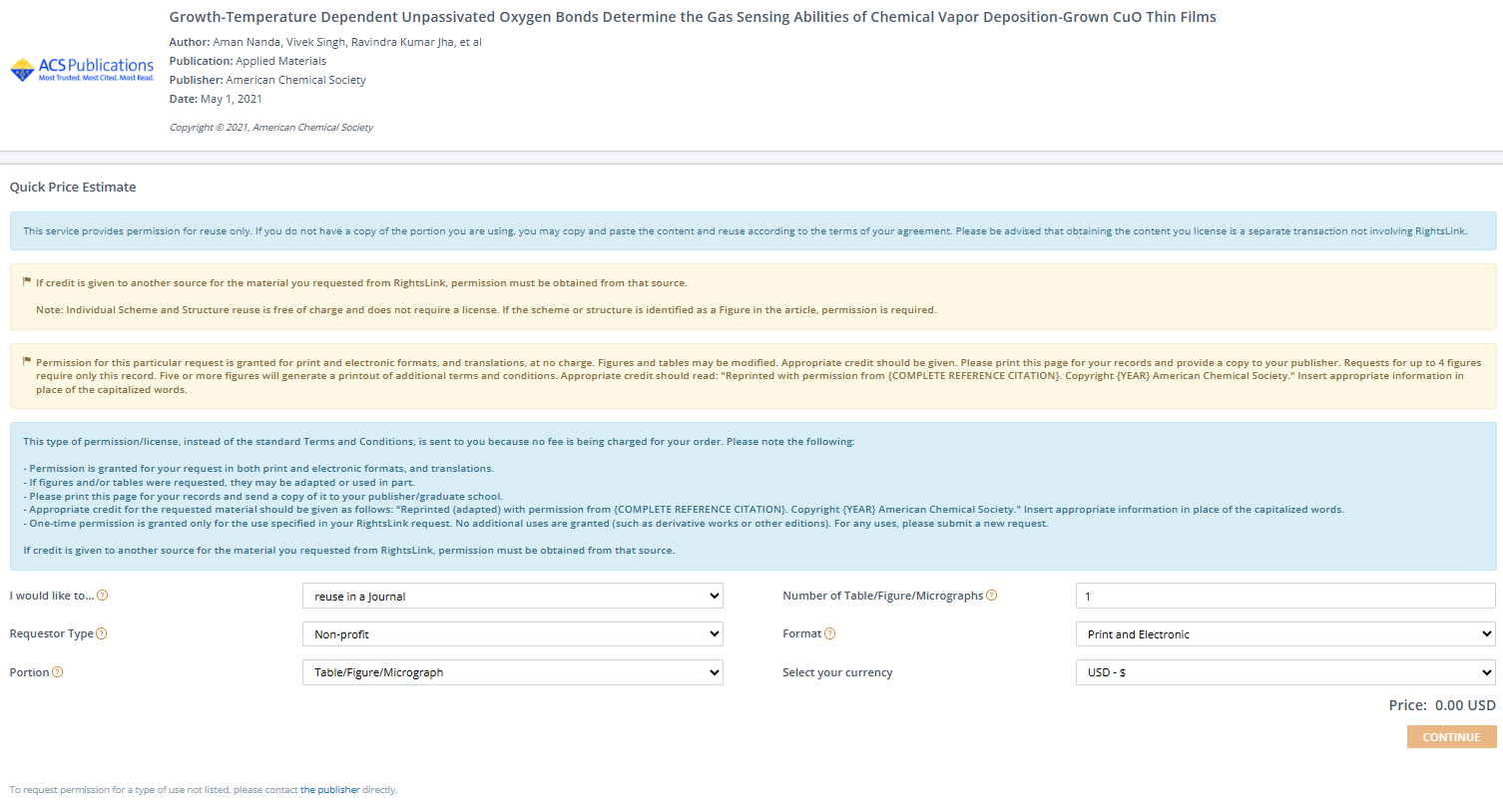


**Fig. 4a**

**
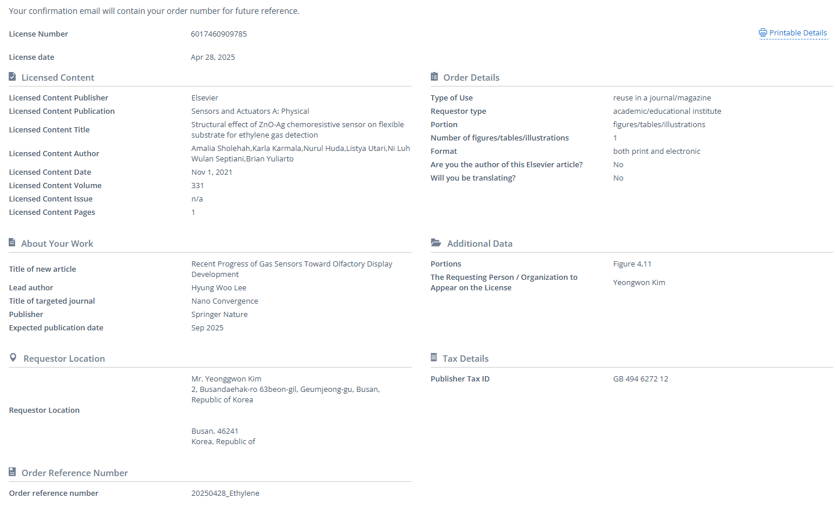
**

**Fig. 4b**

**
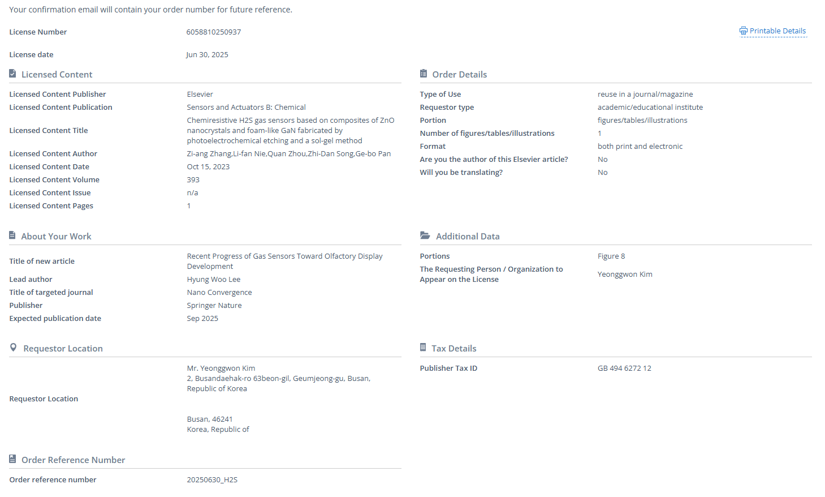
**

**Fig. 4c**

**
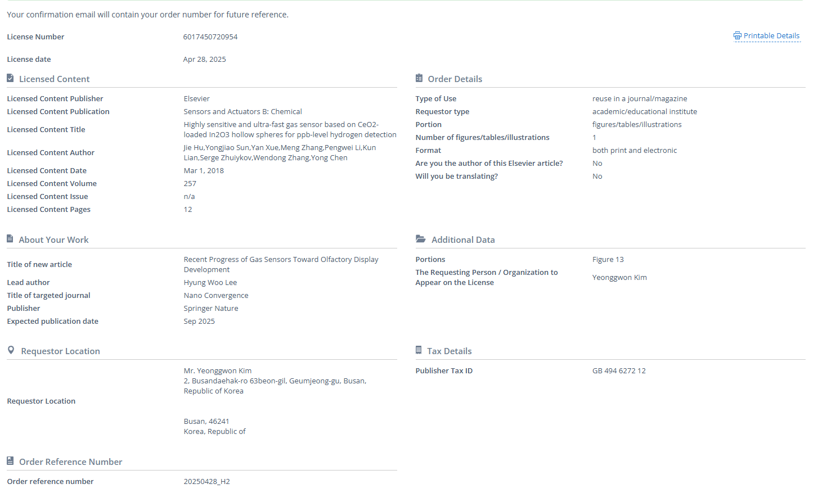
**

**Fig. 4d**

**
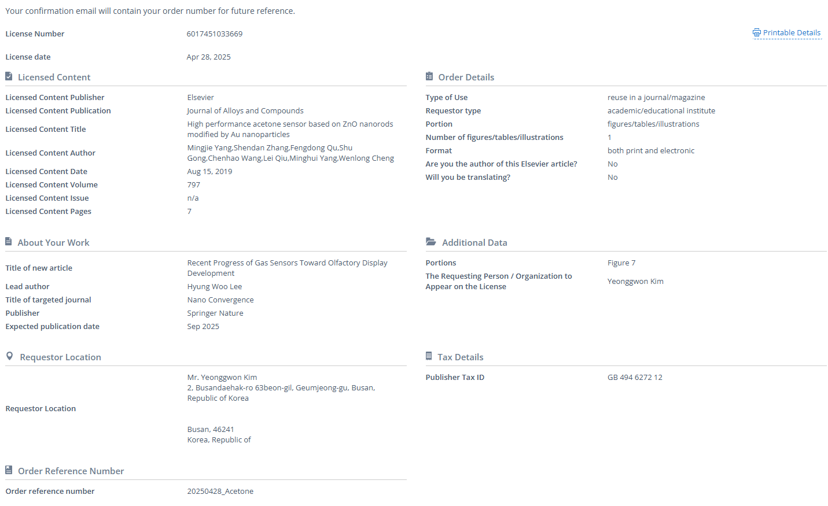
**

**Fig. 4e**

**
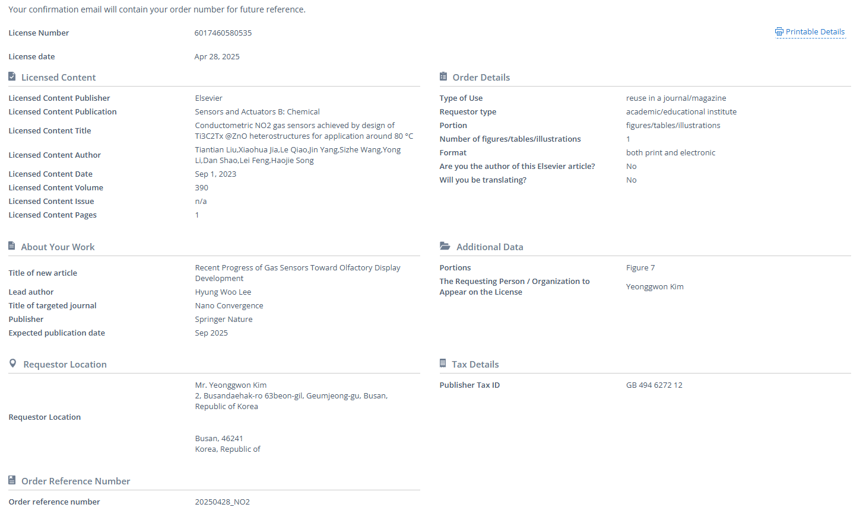
**

**Fig. 5a**


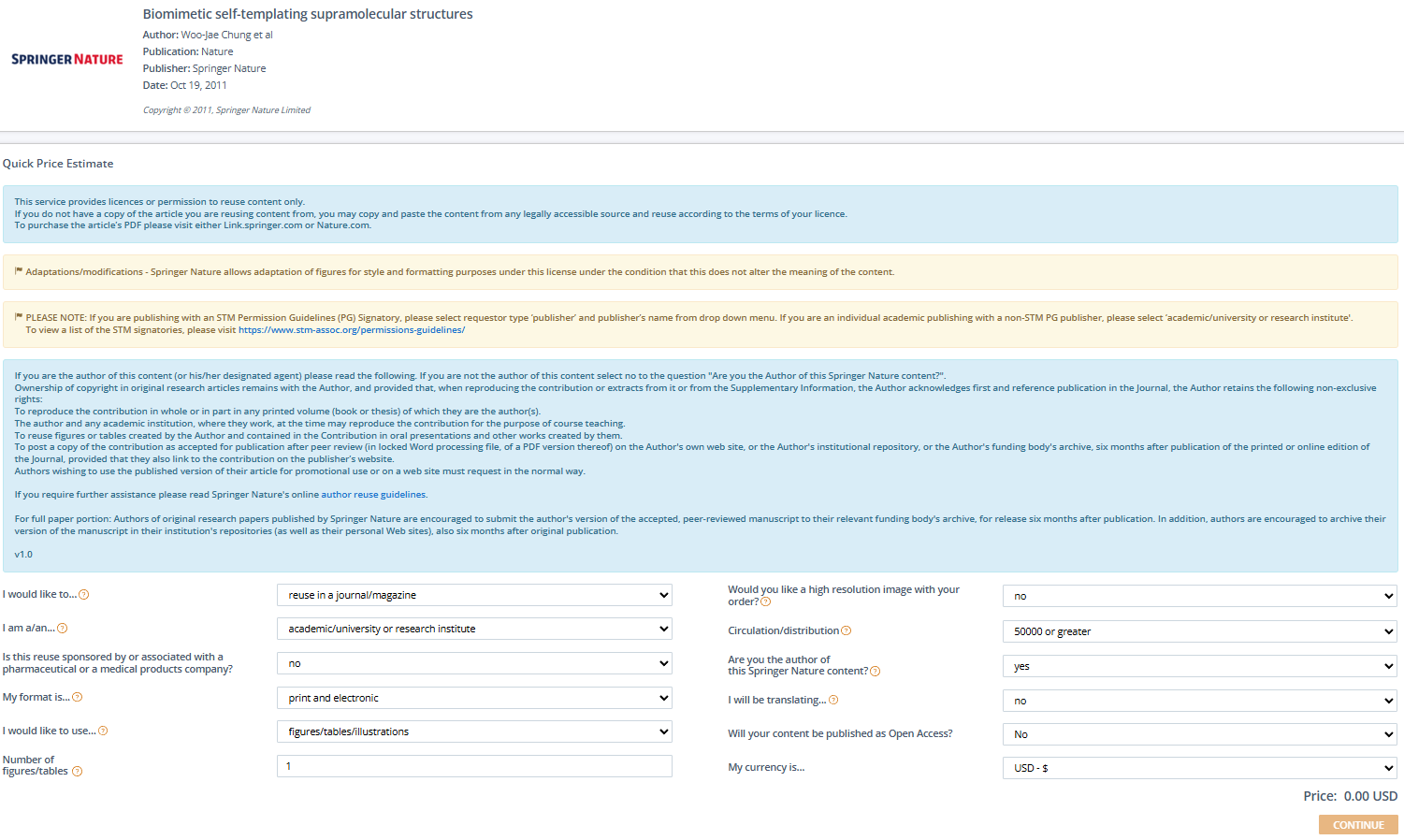


**Fig. 5b**


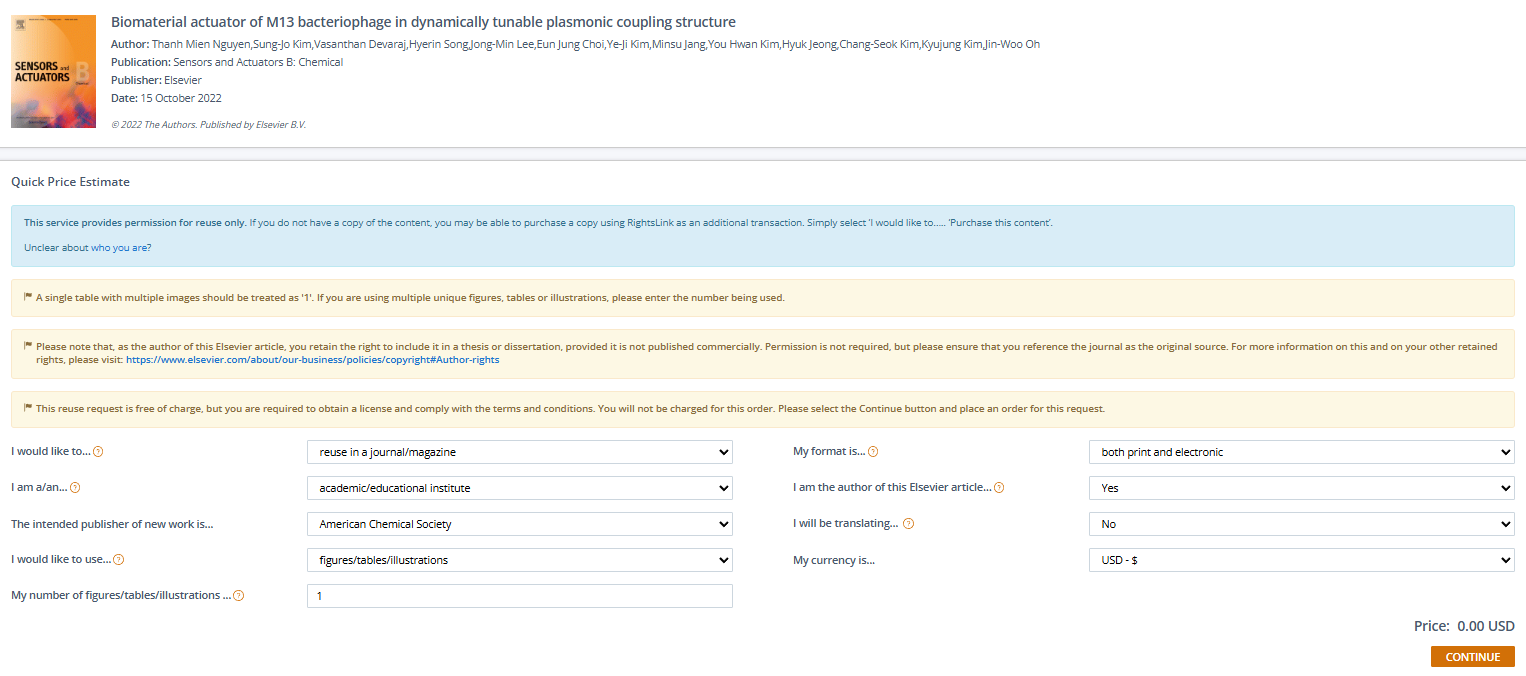


**Fig. 5c**


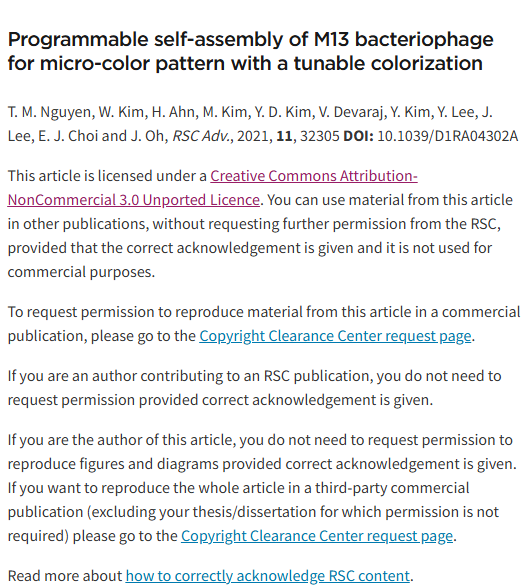


**Fig. 6a**

**
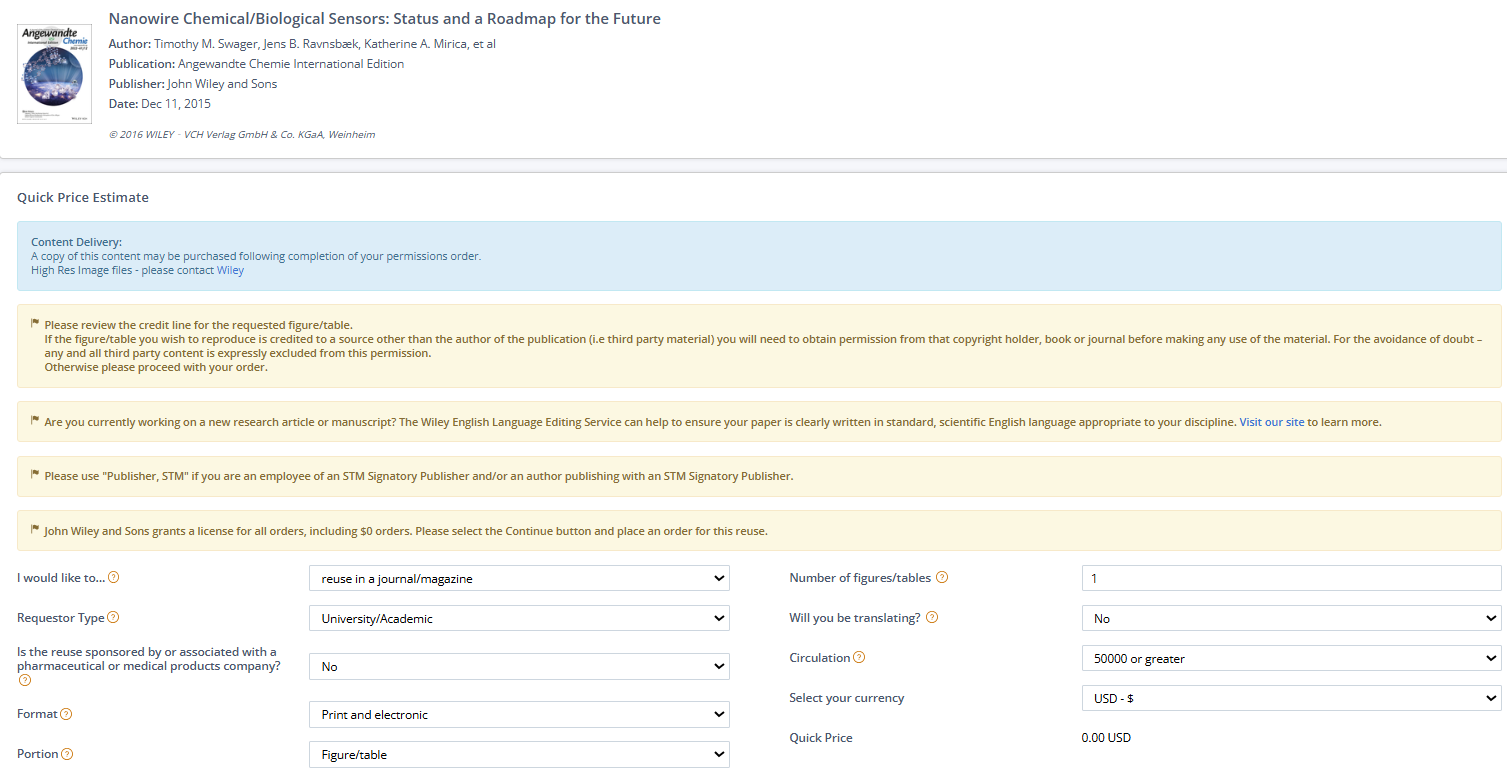
**

**Fig. 6b**

**
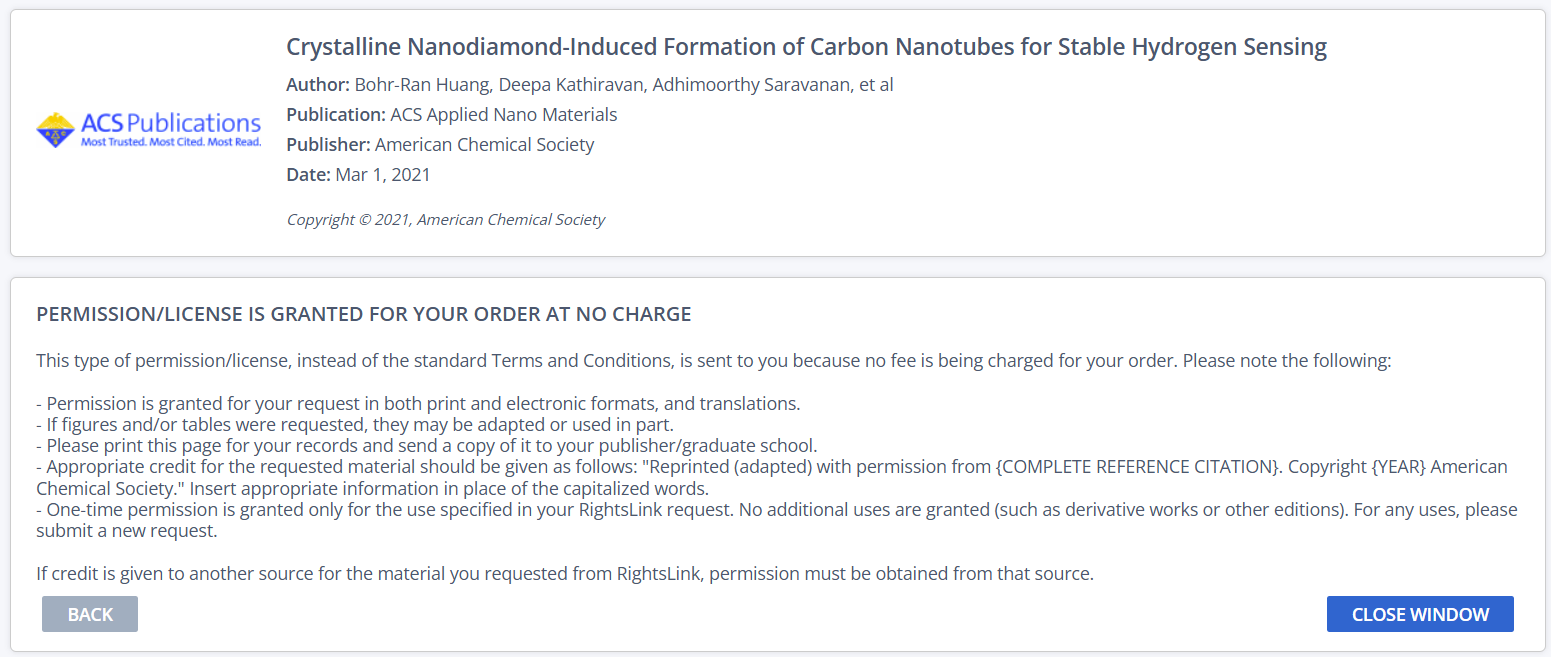
**

**Fig. 6c**

**
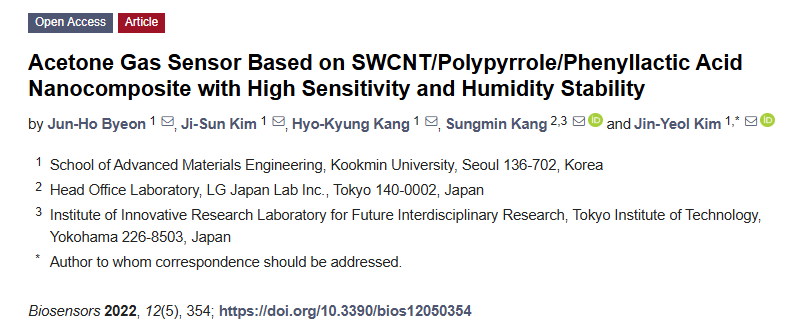
**

**
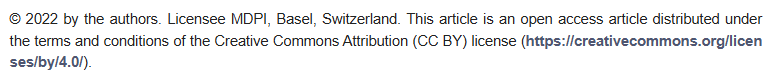
**

**Fig. 6d**

**
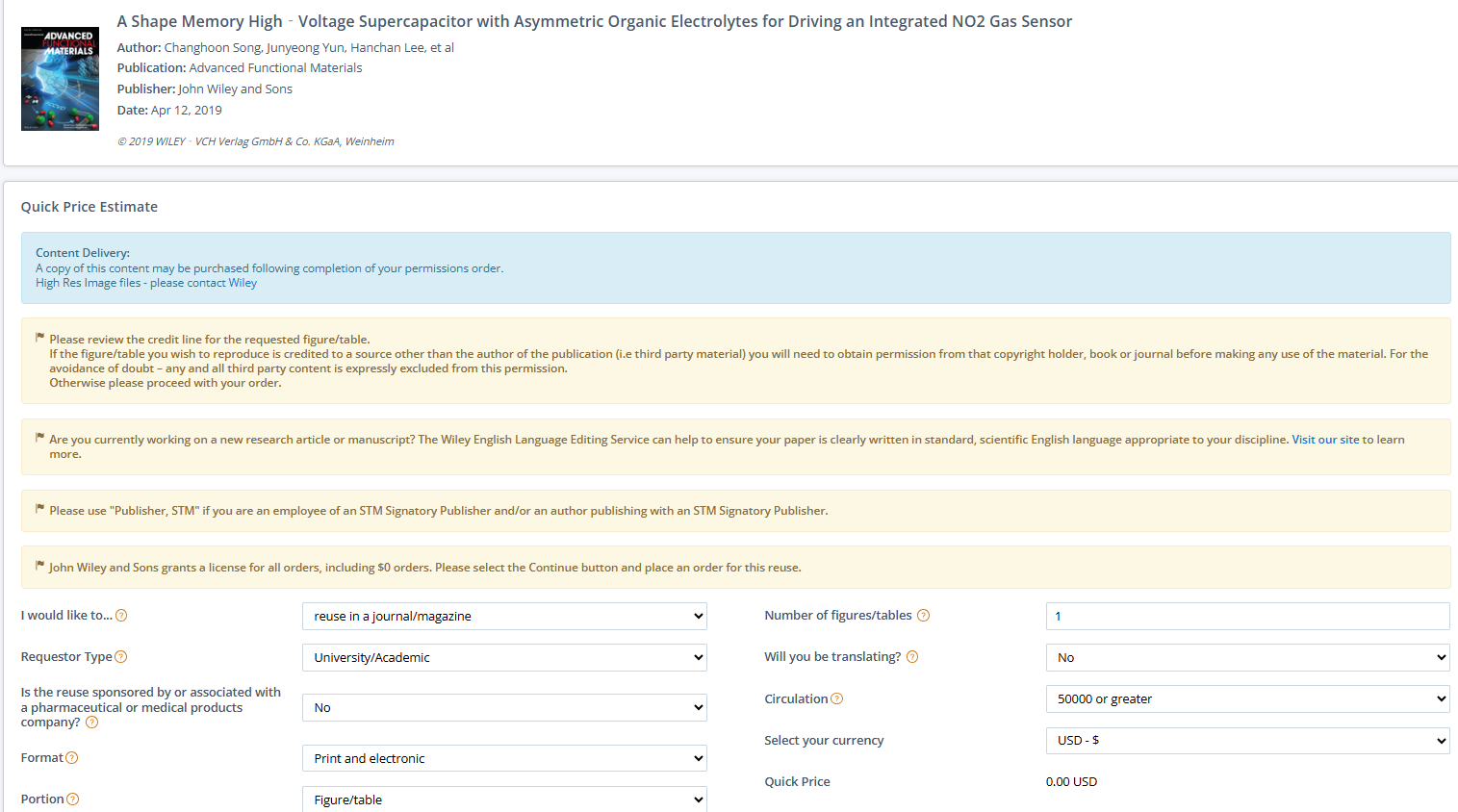
**

**Fig. 6e**

**
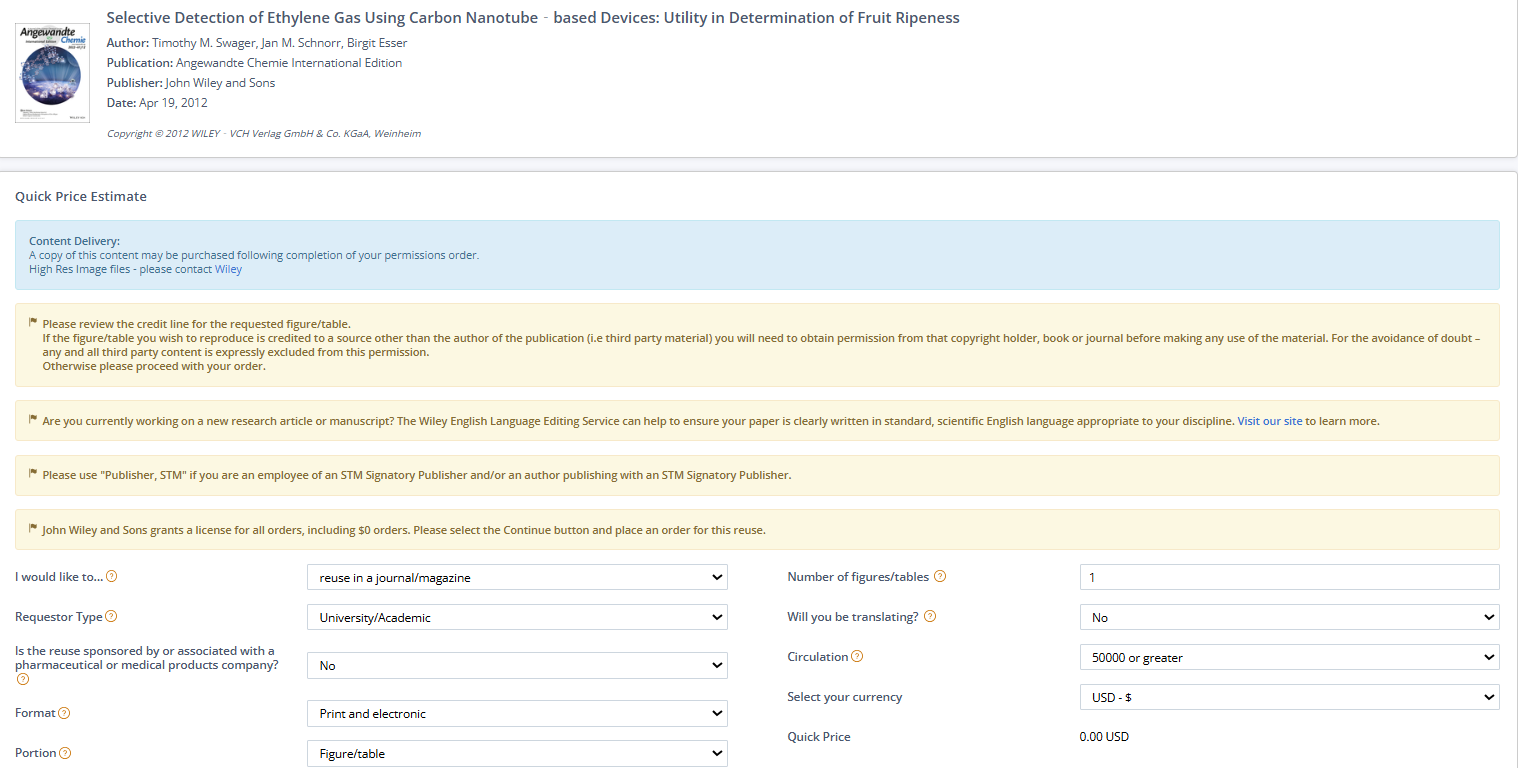
**

**Fig. 6f**

**
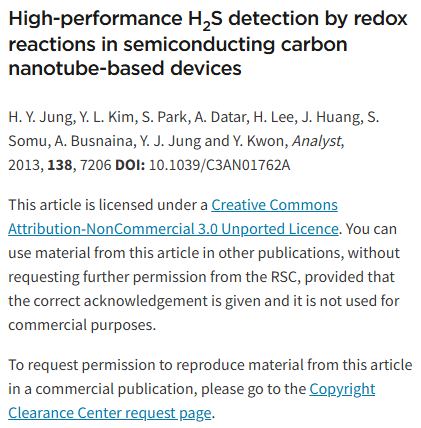
**
